# The contribution of transposable elements to size variations between four teleost genomes

**DOI:** 10.1186/s13100-016-0059-7

**Published:** 2016-02-09

**Authors:** Bo Gao, Dan Shen, Songlei Xue, Cai Chen, Hengmi Cui, Chengyi Song

**Affiliations:** Institute of Epigenetics & Epigenomics, College of Animal Science & Technology, Yangzhou University, Yangzhou, Jiangsu 225009 China

**Keywords:** Transposable elements, Teleosts, Genome size evolution, Activity, Diversity

## Abstract

**Background:**

Teleosts are unique among vertebrates, with a wide range of haploid genome sizes in very close lineages, varying from less than 400 mega base pairs (Mb) for pufferfish to over 3000 Mb for salmon. The cause of the difference in genome size remains largely unexplained.

**Results:**

In this study, we reveal that the differential success of transposable elements (TEs) correlates with the variation of genome size across four representative teleost species (zebrafish, medaka, stickleback, and tetraodon). The larger genomes represent a higher diversity within each clade (superfamily) and family and a greater abundance of TEs compared with the smaller genomes; zebrafish, representing the largest genome, shows the highest diversity and abundance of TEs in its genome, followed by medaka and stickleback; while the tetraodon, representing the most compact genome, displays the lowest diversity and density of TEs in its genome. Both of Class I (retrotransposons) and Class II TEs (DNA transposons) contribute to the difference of TE accumulation of teleost genomes, however, Class II TEs are the major component of the larger teleost genomes analyzed and the most important contributors to genome size variation across teleost lineages. The hAT and Tc1/Mariner superfamilies are the major DNA transposons of all four investigated teleosts. Divergence distribution revealed contrasting proliferation dynamics both between clades of retrotransposons and between species. The TEs within the larger genomes of the zebrafish and medaka represent relatively stronger activity with an extended time period during the evolution history, in contrast with the very young activity in the smaller stickleback genome, or the very low level of activity in the tetraodon genome.

**Conclusion:**

Overall, our data shows that teleosts represent contrasting profiles of mobilomes with a differential density, diversity and activity of TEs. The differences in TE accumulation, dominated by DNA transposons, explain the main size variations of genomes across the investigated teleost species, and the species differences in both diversity and activity of TEs contributed to the variations of TE accumulations across the four teleost species. TEs play major roles in teleost genome evolution.

**Electronic supplementary material:**

The online version of this article (doi:10.1186/s13100-016-0059-7) contains supplementary material, which is available to authorized users.

## Background

TEs are mobile genetic units and are a major constituent of a cell’s “mobilome”. They exhibit a broad range of diversity in their structure and transposition mechanisms, and are subdivided into two classes depending on their transposition mode: via RNA for class I retrotransposons and via DNA for class II transposons [1]. Class I retrotransposons include long terminal repeat retrotransposons (LTRs), long interspersed nuclear elements (LINEs), and short interspersed nuclear elements (SINEs) [2]. Class II transposons can be divided into three major subclasses: cut-and-paste DNA transposons, rolling-circle DNA transposons (Helitrons), and self-synthesising DNA transposons (Polintons/Mavericks) [3]. Cut-and-paste transposons, which are very diverse, have been classified into superfamilies (hAT, Tc1/Mariner, etc.) based on the similarity of their transposases and on shared structural features, including the terminal inverted repeat (TIR) sequence and the length of the target site duplication (TSD) that flanks the TIR and is generated during integration [3]. Due to their unique ability to transpose, and because they frequently amplify, TEs are major determinants of genome size [4, 5] and have been highly influential in shaping the structure and evolution of eukaryotic genomes. TEs constitute the largest component of mammalian genomes [6–8]; using the RepeatMasker approach [9, 10] it was predicted that approximately half of the human genome is covered by TEs, while recent annotation by the P-clouds pipeline suggests the TE coverage in human genome may be closer to two-thirds [11]. Most TEs of mammals are belong to class I retrotransposons, and the L1 family of LINEs is still active [6–8, 10].

Teleostean fish constitute the most diverse vertebrate group, and this diversity is also reflected in the diversity of their genome size and structure [12]. Although the available genome sequences for analysis (over 10 species) is minuscule in the huge species diversity of this clade, four representative teleost species, zebrafish (*Danio rerio*, Dr), medaka (*Oryzias latipes*, Ol), stickleback (*Gasterosteus aculeatus*, Ga), and tetraodon (*Tetraodon nigroviridis*, Tn), being of particular interest both experimentally and evolutionarily, have been sequenced as well [13–16]. Medaka, stickleback, and tetraodon belong to the superorder of *Acanthopterygii*, zebrafish belongs to the superorder of *Ostariophysi*; they all arose in the triassic period and are relatively close compared with the other class fishes [17]. However, genome sizes vary across these four teleost species by over four times. The zebrafish genome, with a size of approximately 137.17 Mb, is the largest, followed by the medaka with 869.00 Mb, then the stickleback with 461.53 Mb, while the tetraodon genome, with 358.62 Mb, is the smallest [12]. The variation of genome size between these close lineages remains largely unexplained. Transposable elements (TEs), as a major component of vertebrate genomes, may be a potential source for understanding the fish genome evolution. The initial annotations of four teleost (zebrafish, medaka, stickleback, and tetraodon) genomes have suggested that major differences in TE content exist between lineages [13–16]; and comparisons of TE diversity and evolution have revealed that teleost genomes contain the highest diversity of TE superfamilies in vertebrates [18], however, the TE contents in the early assembles of medaka, stickleback, and tetraodon tent to be underestimated and inaccurate due to the repeat database is far from complete; information on the distribution of TE diversity and density, and the evolution dynamics intra-species of teleosts, and the knowledge of the roles of TEs in teleost genome architecture and evolution is still reduced and fragmented. To better understand the different success rates of TEs and the evolution of genomes within teleosts, in this study we re-annotated the mobilomes of four representative teleost species (zebrafish, medaka, stickleback, and tetraodon) by using multiple de novo repeat prediction pipelines (RepeatModel, MGEScan-non-LTR, LTRharvest, RetroTector) with a combination of known repeat elements from the RepBase database; we identified diverse autonomous families of DNA transposons (hAT and Tc1 superfamilies) and retrotransposons, investigated the evolutionary pattern of TEs and the phylogenetic relationship among various TE clades and superfamilies, and highlighted the differences of TE activity, diversity and abundance within four teleost species. By integrating analyses of these four teleost species, we can perform a comprehensive analysis of mobilomes across the four species and make inferences about the causes of genome size variations within the four teleosts.

## Results

### Dramatically different expansion of TEs across the four teleost genomes

**Fig. 1:**
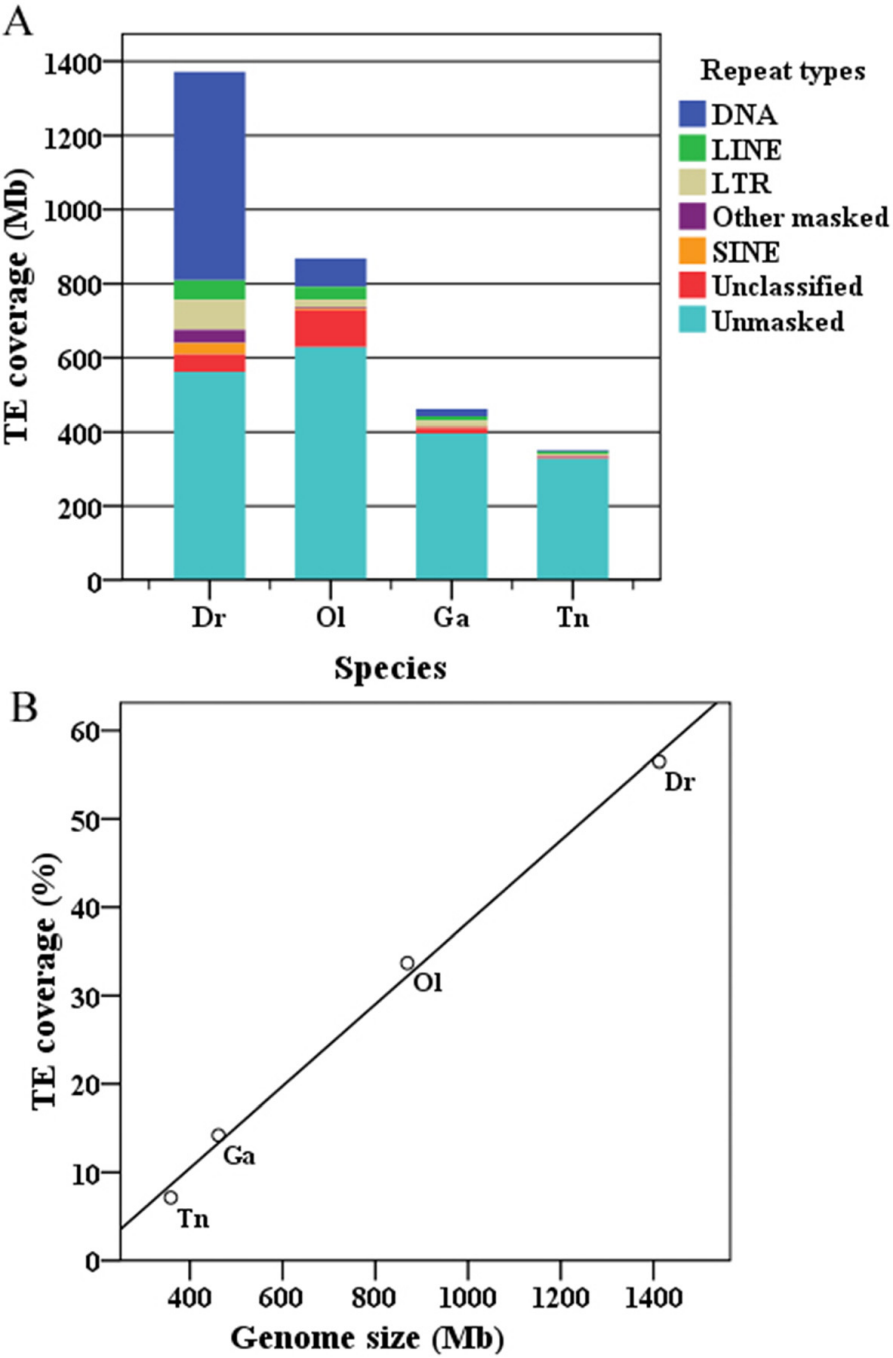
Genome size and TE coverage in teleost genomes. **a** TE coverage in the four teleost species; (**b**) correlation analysis between the variations in genome size and TE content. Genome sizes were plotted against percentages of TE coverage for four teleost species. The *black line* represents the linear regression of the plot

The joint annotation of teleost mobilomes with the species-specific custom TE libraries, which combined the previously-known elements from RepBase and the elements newly identified by multiple de novo methods as described in the Methods section, revealed a significantly different expansion of TEs within four teleost species (Table 1 and Fig. 1). The largest genome, that of the zebrafish, shows a dramatic accumulation of TEs, and the total interspersed repeats comprise over half of the sequenced genome (56.49 %/773.70 Mb). This is highest of the four investigated teleost species, followed by the medaka (33.70 %/236.28 Mb), and the stickleback (14.21 %/63.48 Mb). In the smallest genome, that of the tetraodon, which also represents the most compact genome described in vertebrates, the repeat content only represents 7.13 % (21.55 Mb) of the genome (Table 1 and Fig. 1a). The variation of genome size correlates with TE contents across the four teleost species (Fig. 1b). Our data clearly shows that differential accumulations of TEs contributed to the size variation of the four teleost genomes.

**Table 1:** TE coverage in teleost genomes^a^

	Zebrafish		Medaka		Stickleback		Tetraodon	
	Count	(%/Mb)	Count	(%/Mb)	Count	(%/Mb)	Count	(%/Mb)
Total retrotransposons	533112	12.00/164.29	215153	8.37/58.71	105276	6.61/29.50	35607	4.00/12.08
SINE	136879	2.24/30.64	30578	0.68/4.79	11523	0.67/2.97	1498	0.09/0.26
LINE	132888	3.85/52.78	112487	4.97/34.86	35604	2.60/11.61	19385	1.97/5.94
LTR	160149	5.90/80.87	72088	2.72/19.05	58159	3.34/14.92	14724	1.95/5.89
DNA	2368307	41.07/562.49	282359	11.00/77.14	73571	4.47/19.96	21901	1.55/4.68
Unclassified	228249	3.43/46.92	397468	14.32/100.42	72717	3.14/14.02	18465	1.58/4.78
Total interspersed repeats		56.49/773.70		33.70/236.28		14.21/63.48		7.13/21.55
Small RNA	13817	0.12/1.65	7223	0.16/1.09	2950	0.10/0.45	784	0.04/0.13
Satellite	75515	1.50/20.61	3046	0.16/1.13	1309	0.09/0.41	560	0.08/0.23
Simple repeats	42321	0.99/13.50	15283	0.29/2.03	8876	0.25/1.12	22873	0.74/2.25
Low complexity	1128	0.03/0.35	149	0.00/0.03	243	0.01/0.04	300	0.02/0.05

^a^The custom library combined with the repeats from RepBase (version 20150807) and de novo repeats was used for the all investigated teleost genomes

The greatest difference in TEs between the teleost species lies in the abundance of class II TEs (DNA transposons; Table 1 and Fig. 1a). This class of repeats has a striking amplification in the largest genome of zebrafish, where they contribute over 41.07 % (562.49 Mb) of the sequenced genome. In the second largest genome, the medaka, DNA repeats contribute 11.00 % (77.14 Mb) of the genome (Table 1). However, the proliferation of DNA transposons in the smaller genomes of the stickleback and tetraodon is weak, and this class of TEs only represents 4.47 % (19.96 Mb) and 1.55 % (4.68 Mb) of their sequenced genomes, respectively (Table 1). Retrotransposons (class I transposons), including SINE, LINE and LTR repeats, also display different expansions between teleost species. The overall contents for retrotransposons represent 12.00 % (164.29 Mb) of the zebrafish genome, which is substantially higher than that in the medaka (8.37 %/58.71 Mb), stickleback (6.61 %/29.50 Mb), and tetraodon (4.00 %/12.08 Mb) genomes; the zebrafish represents the highest abundance of both LTR (5.90 %) and SINE (2.24 %) retrotransposons across teleost species; while the medaka shows the highest accumulation of LINEs at 4.97 % of the total sequenced genome (Table 1). Compared with other types of TEs, SINEs represent a relatively weak proliferation in most teleost species except zebrafish (Table 1). The proportion of satellites in the zebrafish genome (1.50 %) is higher than that observed in the medaka (0.16 %), stickleback (0.09 %), and tetraodon (0.08 %) genomes. The proportion of simple repeats in the zebrafish genome (0.99 %) is higher than that in the tetraodon (0.74 %), medaka (0.29 %) and stickleback (0.25 %) genomes (Table 1).

### Dramatically different accumulation of DNA transposons across the four teleost genomes

**Fig. 2:**
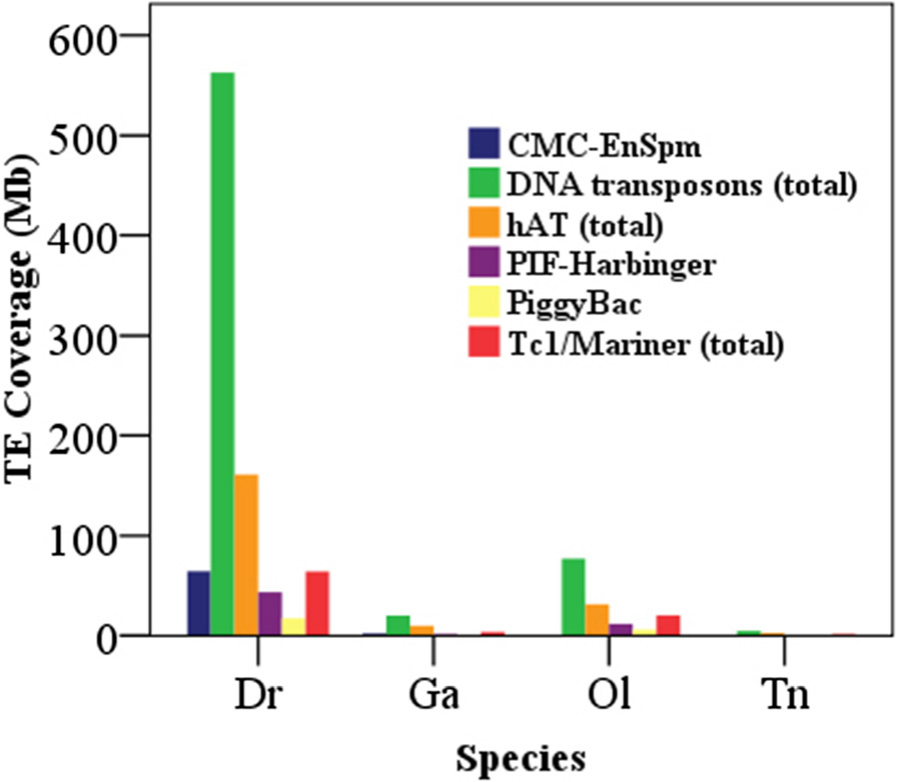
Distribution and abundance of DNA transposons in four species of teleost

A comparison of the diversity and abundance distributions of DNA TEs across the four teleost genomes revealed striking differences both between superfamilies and between species (Fig. 2, Table 2, and Additional file 1: Table S1). In total, 19 superfamilies of Class II transposons, representing all three main types of DNA transposons (cut-and-paste, rolling-circle, and self-synthesising) were detected in the four teleost genomes (Additional file 1: Table S1), and the results of abundance distribution of DNA repeats are summarized in Table 2. Among the three main types of DNA transposons, both rolling-circle (Helitron) and self-synthesising (Mavericks) DNA transposons were detected within the four teleost genomes with the absence of self-synthesising (Mavericks) DNA transposon in the medaka; these two superfamilies represent much lower abundance within these teleost species, with less than 0.2 % genome coverage, with the exception of Helitron, which has greater expansion in the zebrafish genome and contributes 1.42 % (19.54 Mb) to the genomic sequence (Table 2). The diversity of repeat types of cut-and-paste DNA transposons observed at the level of the superfamily in the zebrafish (18), medaka (14) and stickleback (13) genomes is broadly similar, while the tetraodon genomes contains reduced superfamilies; several superfamilies of cut-and-paste DNA transposons, including Academ, Kolobok, MULE-MuDR, PIF-ISL2EU, and Sola, observed in the other three teleost species, are absent in the tetraodon genome (Table 2). At the family level, the larger genomes also appear to have many more non-redundant families in each superfamily than the small ones; totally, 1249, 234, 161, 74 non-redundant families were detected in zebrafish, medaka, stickleback, and tetraodon; Typically, over 16 times more non-redundant families were observed in the zebrafish than in the tetraodon (Additional file 1: Table S1). This indicates that the greater TE content in the lineages of large genome compared with the lineages of small ones is based not only on greater numbers of elements but also on greater element diversity at the more fine-scale family level.

**Table 2:** Abundance of DNA transposons in teleost genomes

Type/superfamily/family	TE coverage (copy number/base pairs masked/%)			
	Zebrafish	Medaka	Stickleback	Tetraodon
Cut and paste TE				
Academ	987/87534/0.01	791/145168/0.02	334/48465/0.01	
CMC-Chapaev-3		42/21183/0.00		
CMC-EnSpm	382820/64375022/4.69	162/35330/0.00	5353/1984708/0.43	1524/119135/0.03
Crypton	36092/4088708/0.30	8275/1835625/0.21		
Dada	17226/2082682/0.15	549/121682/0.01	118/44503/0.01	936/109835/0.03
Ginger	6053/551259/0.04	236/55011/0.01		
IS3EU	17838/2815133/0.21			
Kolobok	128817/33082060/2.41	14/2093/0.00	583/74420/0.02	
Merlin	76084/8473015/0.62		710/437407/0.09	
MULE-MuDR	4276/2180335/0.16	206/46981/0.01	70/16079/0.00	
MULE-NOF	1159/272049/0.02			
P	7170/1900925/0.14			
PIF-Harbinger	110931/43436661/3.17	32217/11657322/1.34	3030/1513874/0.33	539/106002/0.03
PIF-ISL2EU	2262/959233/0.07	216/157939/0.02	365/213849/0.05	
PiggyBac	45457/17290031/1.26	34982/6161692/0.71	890/125960/0.03	359/119750/0.03
Sola	15251/3243347/0.24	1245/325083/0.04	419/220972/0.05	
Zisupton	6559/1093949/0.08			
Tc1/Mariner (total)	192070/64157597/4.68	65677/20436282/2.35	9784/3576109/0.78	1401857/0.39
Tc1	122464/47220045/3.44	37350/13303937/1.53	6236/2542455/0.55	1578/513677/0.14
pogo	5844/1778814/0.13	27376/6703781/0.77	2439/635035/0.14	2781/832008/0.23
Other families	4632/1895302/0.14	951/428564/0.05	1109/398619/0.09	200/56172/0.02
Unclassified Tc1/Mariner	59130/13263436/0.97			
hAT (total)	708715/160965573/11.73	116937/31365783/3.61	43229/9730722/2.11	12659/2713799/0.75
Ac	222707/57824458/4.22	29754/7436916/0.86	23961/3811137/0.83	1243/339847/0.09
Charlie	133312/26390661/1.92	72169/20423089/2.35	11098/4133497/0.90	7615/1794070/0.50
Other families	97917/19890417/1.45	7404/1871492/0.22	3074/511394/0.11	1211/362560/0.10
Unclassified hAT	254779/56860037/4.15	7610/1634286/0.19	5096/1274694/0.28	2590/217322/0.06
Self-synthesizing TE				
Maverick	11019/1934799/0.14		538/558181/0.12	1243/101677/0.03
Rolling-circle TE				
Helitron	105567/19541897/1.42	3043/304374/0.04	1564/204360/0.04	187/50445/0.01

These DNA transposons dominate the size variation in teleost genomes; the larger genomes accumulate many more DNA repeats than smaller ones. Typically, over 100 times more genome content (562.49 Mb) derived from DNA transposon amplification was identified in the zebrafish than in the tetraodon (4.68 Mb), and almost all types of DNA repeats appear to occur more frequently in the larger genomes than the smaller ones (Tables 1 and 2). Two dominant families of cut-and-paste DNA transposons in all four teleost species are hAT and Tc1/Mariner (Table 2). Four of the other cut-and-paste DNA superfamilies (CMC-EnSpm, PIF-Harbinger, Kolobok, and PiggyBac) have also amplified to significant numbers (over 1 %) in the zebrafish genome. In addition to hAT and Tc1/Mariner, the PIF-Harbinger superfamily in the medaka genome has amplified to significant numbers as well, and comprised 1.34 % (11.66 Mb) of the genomic sequences. The other superfamilies did not show significant expansion (<1 %) in the four teleost genomes (Fig. 2 and Table 2).

**Fig. 3:**
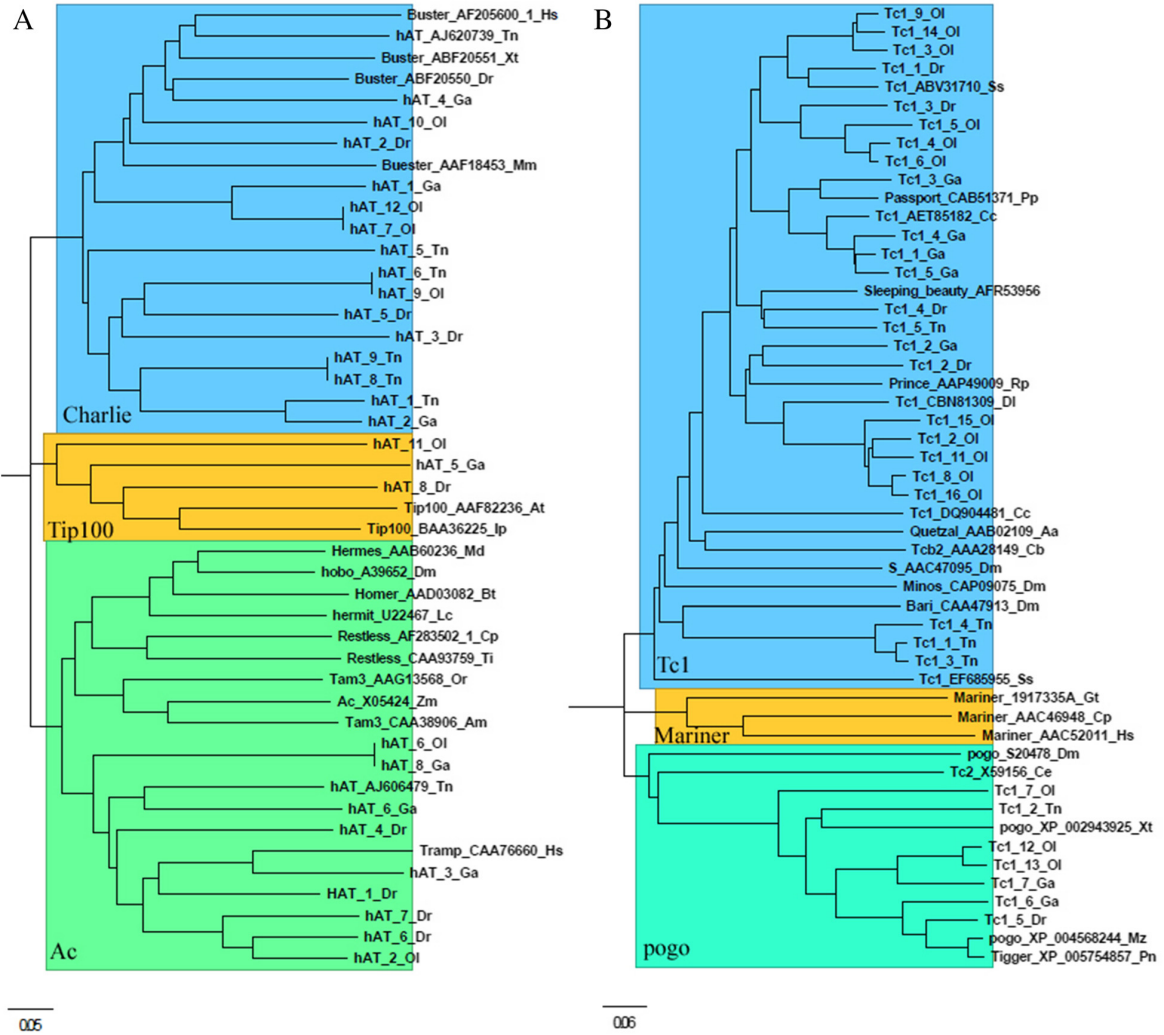
Phylogenetic position of hAT (**a**) and Tc1/Mariner (**b**) in teleost genomes relative to previously described families

The hAT is the most abundant and diverse DNA transposon superfamily, represented by multiple families in all four teleost genomes (Ac, Charlie, Tip100, Tol2, hobo etc.) (Additional file 1: Table S1), which contributes 11.73 % (160.97 Mb), 3.61 % (31.36 Mb), 2.11 % (9.73 Mb), and 0.75 % (2.71 Mb) to the zebrafish, medaka, stickleback, and tetraodon genomes, respectively (Fig. 2 and Table 2). Seven, 7, and 5 autonomous subfamilies of hAT in medaka, stickleback, and tetradodon identified by TBLAST program (Additional file 1: Table S1), were combined with the eight autonomous subfamilies of hAT in zebrafish from RepBase to build the Phylogenetic tree. And the phylogenetic analysis of the hAT autonomous subfamilies with known reference elements revealed that these autonomous hAT subfamilies were classified into the Ac, Charlie, and Tip100 families, and majority of them belong to Ac and Charlie families, only one Tip100 family was detected in zebrafish, medaka, and stickleback, respectively (Fig. 3a). The Tc1/Mariner is the second most abundant DNA transposon superfamily in the teleost genomes, and contains diverse families (Tc1, pogo, ISRm11, Stowaway etc.) (Additional file 1: Table S1), and comprises 4.68 % (64.16 Mb), 2.35 % (20.44 Mb), 0.78 % (3.58 Mb), and 0.39 % (1.40 Mb) of the zebrafish, medaka, stickleback, and tetraodon genomes, respectively (Fig. 2 and Table 2). Five, 14, 7, and 5 new autonomous subfamilies of Tc1/Mariner superfamily were extracted in zebrafish, medaka, stickleback, and tetradodon (Additional file 1: Table S1), respectively, and used for the phylogenetic analysis. Phylogenetic tree revealed all autonomous Tc1/Mariner transposons in teleosts belong to the Tc1 and pogo families, majority of them belong to Tc1 family, and few of them were classified as pogo family, no autonomous Mariner transposon was detected in all four teleosts (Fig. 3b).

**Fig. 4:**
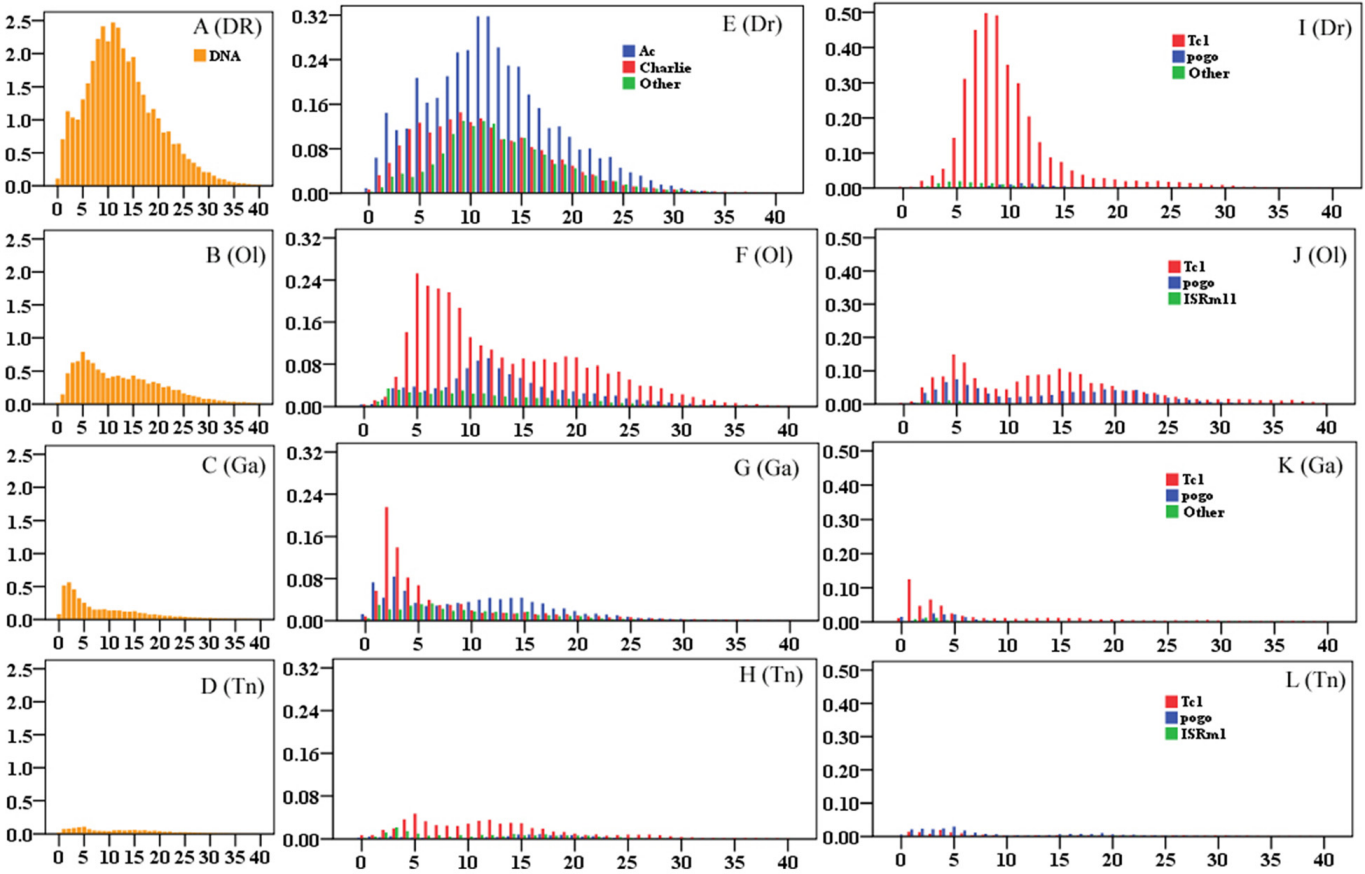
Divergence distribution of DNA transposons (**a**-**d**), hAT (**e**-**h**), and Tc1/Mariner (**i**-**l**) superfamilies in the zebrafish (**a**, **e**, **i**), medaka (**b**, **f**, **j**), stickleback (**c**, **g**, **k**), and tetradon (**d**, **h**, **l**) genomes. The x-axis represents the substitution rate from consensus sequences (%), and the y-axis represents the percentage of the genome comprised of repeat classes (%)

Comparison of the sequence divergence distribution of DNA TEs revealed an extraordinary difference of proliferation dynamics across the four teleost genomes (Fig. 4). Overall, the DNA transposons within the largest genome of zebrafish have been active over a longest time period, and exhibited a strongest activity during the evolution history compared with other teleost, as shown by the broadest distribution of divergence ranging from 0 to 35 % and a very sharp peak of divergence at about 10 % (Fig. 4a). In contrast, the accumulation of DNA transposons in both of medaka and stickleback lineages is much weaker than that in zebrafish lineage, and tends to be very recent, with peaks of divergence less than 5 % and striking lacks of ancient proliferation (Fig. 4b and c). While the tetraodon lineage exhibits an extremely low level of activity of DNA TEs (Fig. 4d). In-deep analysis revealed that families in both the hAT and Tc1/Mariner superfamilies display dramatically differential accumulations during their evolutionary histories as well. The dominant families of hAT in teleost genomes are Charlie and Ac; whereas the dominant families of Tc1/Mariner in teleost genomes are Tc1 and pogo (Table 2). Both Charlie and Ac families in the zebrafish and medaka genomes have undergone one round of substantial accumulation between the divergence of 5 and 15 %, followed by a decrease in recent activity (Fig. 4e and f), while the predominantly recent activities of Charlie and Ac families were observed in the stickleback genome in contrast with the very weak activity of these families in the tetraodon genome (Fig. 4g and h). Tc1 family in zebrafish has undergone one round of sharp burst at the divergence of 8 %, followed by recent decrease in activity and dominates the evolution of Tc1/Mariner superfamilies in this lineage; while both Tc1 and pogo families in the medaka genome have undergone two rounds of weak expansion in the evolution histories. Tc1 in stickleback has undergone one round of recent proliferation; the activities of pogo in stickleback, and Tc1 and pogo families in tetraodon are very low in the whole evolution histories (Fig. 4i, j, k and l). Both hAT and Tc1/Mariner superfamilies in some teleost genomes contain active families as shown by the distribution of many elements with <5 % divergence from the consensus (Fig. 4e-k).

### Different distribution of LINE and LTR family diversity within the four teleost genomes

To characterize the family distribution of LINEs in the four teleost species, we applied the MGEScan-non-LTR program [19] to extract the LINE elements. In total, 1324, 436, 188, and 51 ‘ORF-preserving’ LINEs were identified in the genomes of the zebrafish, medaka, stickleback, and tetraodon, respectively. The elements with a long ORF2 (>700aa) and intact RT domain were retained and designated as autonomous LINEs. These newly-identified LINEs were combined with the known autonomous LINEs (ORF2>700aa and intact RT domain) from RepBase, and classified into families based on amino acid sequence similarity (80 %) of ORF2 and the structure of ORFs (Additional file 2: Table S2). A dramatically different distribution of LINE families within species was found: the zebrafish, representing the most diverse lineage, contains 118 LINE families, while the medaka, stickleback, and tetraodon only contain 8, 11, and 2 LINE families, respectively (Table 3).

**Table 3:** Distribution of LINE families in teleost genomes^a^

Clade/Branch	Zebrafish	Medaka	Stickleback	Tetraodon
Total	118	8	11	2
I	9			
L1	82	6	3	2
Swimmer	45			
Tx1-a	14			
Tx1-b	15			2
Tx1-c	8			
L2	11	1	3	
R2	2	1	1	
Rex	9		2	
RTE	5		2	

^a^The newly identified LINEs by MGEScan-non-LTR programme were combined with known LINEs from RepBase, and the family was built up based on the similarity of amino acid sequence of LINE elements (80 %) and the structure of ORFs

**Fig. 5:**
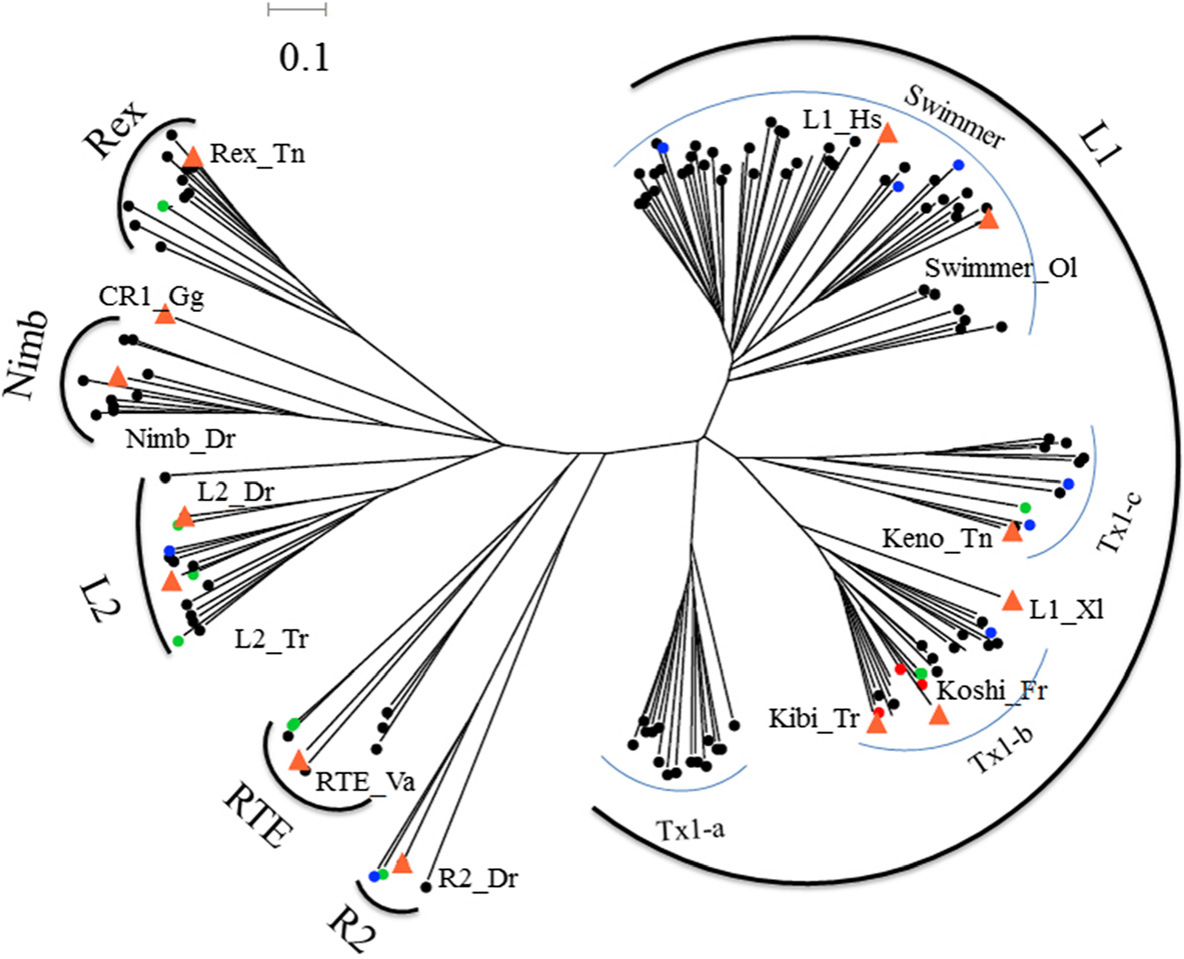
Phylogenetic relationships among the 6 clades of LINEs in the teleost genomes. The nodes of sequences from zebrafish, medaka, stickleback, and tetraodon are shown as *black*, *blue*, *green*, and *red dots*, respectively; and the nodes of reference elements are indicated by *big yellow triangles*. The GenBank accession numbers used for phylogenetic analysis are as follows: Rex_Tn, AJ312227; CR1_Gg, U88211; Nimb_Dr, AL672145; L2_ Dr, AB211150; L2_Tr, AF086712; RTE_Va, AF332697; R2_Dr, AB097126; Kibi_Tr, AB097136; L1_Xl, M26915; Keno_Tn, AB111948; Swimmer_Ol, AF055640; L1_Hs, U93574)

Phylogenetic analysis of these families revealed 6 clades of LINEs in the teleost species (L1, L2, I, Rex-Babar, RTE, and R2), and these clades differ drastically in family diversity among teleost lineages (Table 3 and Fig. 5). The L1 clade is very diverse in the family structure and was further classified into Swimmer, Tx1-a, and Tx1-b and Tx1-c branches, with each branch containing diverse families. The clades of L2, I, and Rex-Babar were less diverse in family structure compared with L1. The R2 and RTE clades had very little diversity in family structure, and only a few families were detected (Table 3 and Fig. 5).

**Fig. 6:**
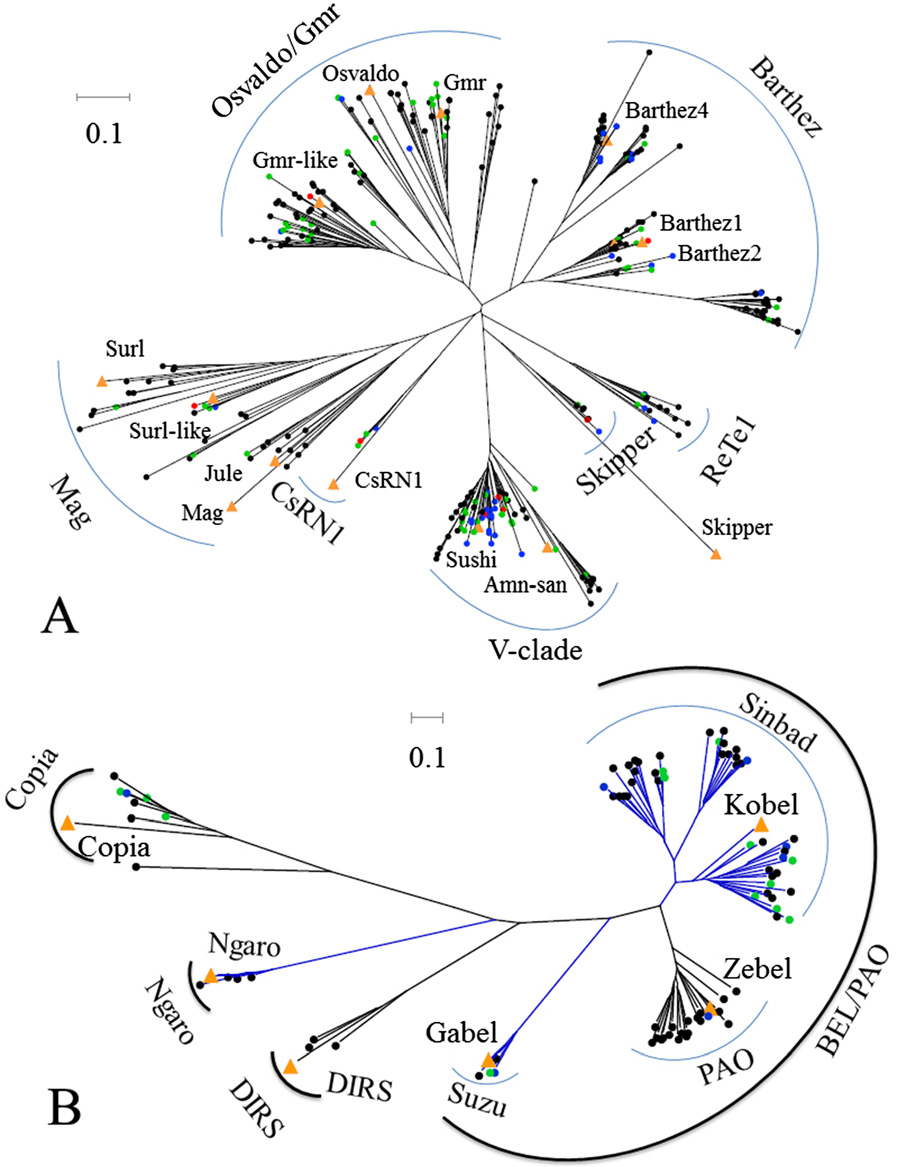
RT phylogenetic tree of Gypsy (**a**) and other groups (**b**) of LTRs in the teleost genomes. The nodes of sequences from the zebrafish, medaka, stickleback, and tetraodon genomes are shown as *black*, *blue*, *green*, and *red dots*, respectively; and the nodes of reference elements are shown with *big yellow triangles*. The GenBank accession numbers used for phylogenetic analysis are as follows: Surl, M75723; Surl-like, AABS01002378; Jule, AY298856; Mag, X17219; CsRn1, AY013571; Sushi, AAC33526; Amn-san, 187466581; Skipper, AF049230; Barthez1, AJ621589; Barthez2, AJ621590; Barthez4, AJ621591; Gmr-like, AJ621595; Gmr, AF104899; Osvaldo, CAB39733; Copia, CAD27357; Ngaro, AAN71721; DIRS, AF442732; Kobel, 154426342; Zebel, 38304119; Gabel, 83921752

**Fig. 7:**
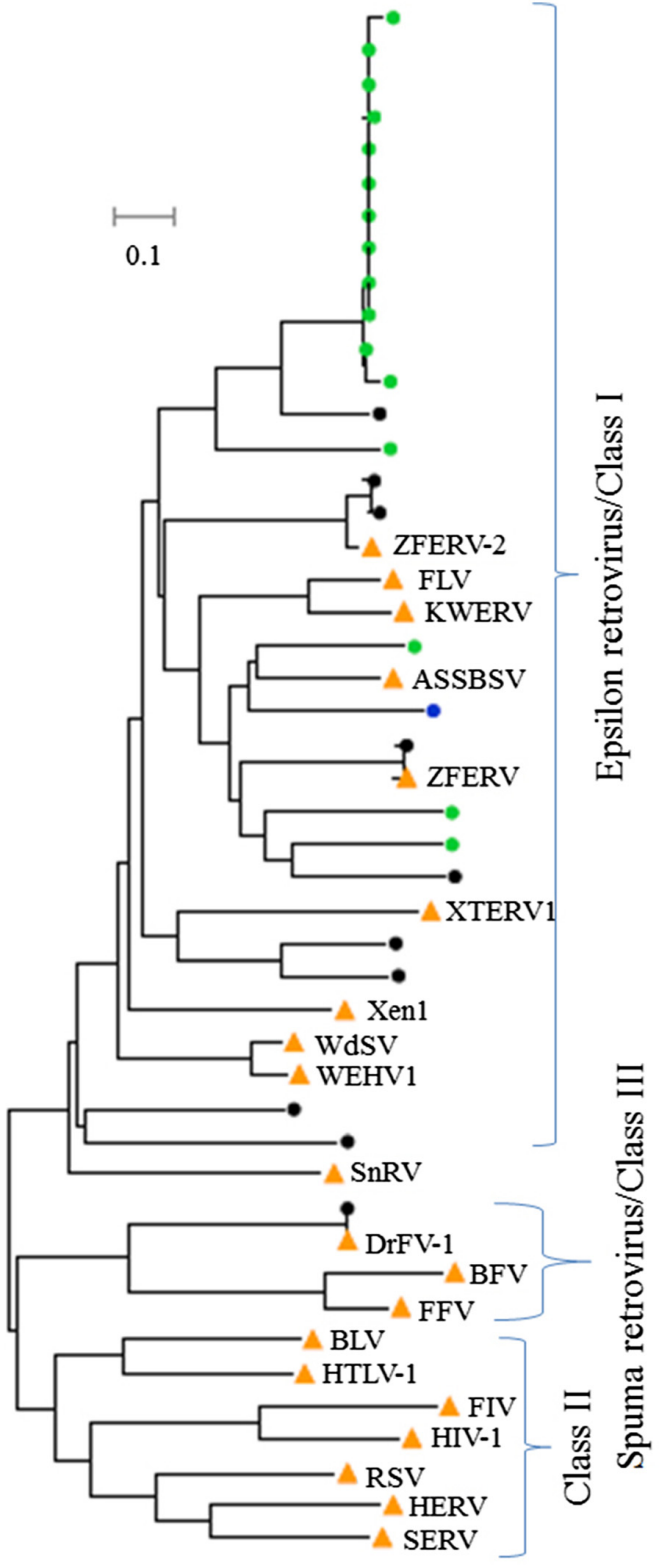
The RT phylogenetic tree of ERVs in teleost genomes. The nodes of sequences from zebrafish, medaka, stickleback, and tetraodon genomes are shown as *green*, *yellow*, *blue*, and *red dots*, and the GenBank accession numbers used for phylogenetic analysis are as follows: ZFERV-2 (Zebrafish endogenous retrovirus 2), 162808041; FLV (Feline leukemia virus), NP_047255; KWERV (killer whale endogenous virus), GQ222416; ASSBSV (Atlantic salmon swim bladder sarcoma virus), ABA54982; ZFERV (Zebrafish endogenous retrovirus), AAM34208; XTERV1 (Xenopus tropicalis endogenous virus 1), HM765512; Xen1 (Xenopus laevis endogenous virus 1), AJ506107; WdSV (Walleye dermal sarcoma virus), AAC82611; WEHV1 (Walleye epidermal hyperplasia virus 1), AAD30048; SnRV (Snakehead fish retrovirus), AAC54861; DrFV-1 (Danio rerio Foamy Virus type 1), 85857417; BFV (Bovine foamy virus), NP_044929; FFV (Feline foamy virus), NP_056914; BLV (Bovine leukemia virus), AAC82587; HTLV-1 (Human T-lymphotropic virus 1), AAC82581; FIV, Feline immunodeficiency virus; HIV-1 (Human immunodeficiency virus 1), AAA43076; RSV (Rous sarcoma virus), BAD98246; HERV (human endogenous retrovirus K10), AAA88033; SERV (Simian endogenous retrovirus), AAC97565

The LTR elements, including ERVs, in the four teleost genomes were extracted using LTRharvest and RetroTector pipelines. The LTR elements with a long ORF (>500aa) and intact RT domain were retained and designated as autonomous LTRs. These LTRs were combined with the known autonomous LTRs (ORF >500aa and intact RT domain) from RepBase, and clustered into LTR families based on amino acid sequence similarity (80 %) (Additional file 3: Table S3). A striking difference in family distribution across species was found; the zebrafish lineage shows an extraordinary diversity of LTRs with 261 LTR families, while the medaka, stickleback, and tetraodon contain only 38, 77, and 8 LTR families, respectively (Table 4). Phylogenetic analysis of these families revealed 6 groups of LTRs (BEL/PAO, Copia, DIRS, Ngaro, Gypsy, and ERV) in teleost species, and these groups differ drastically in family diversity between teleost lineages (Figs. 6 and 7). The Gypsy group is incredibly diverse. In total we identified 7 clades of Gypsy within teleost species by RT phylogenetic analysis (Fig. 6a), six of which correspond to known clades (Gmr/Osvaldo, Barthez, CsRn1, V-calde, Mag, and Skipper), that have been reported in previous reports [20–26]. One new clade (named ReTe1) in teleost species was identified, which doesn’t branch from any of the known reference elements; this clade is close to the Skipper and Barthez clades, but forms a distinct branch. ReTe1 clade distributes in zebrafish, medaka, and stickleback and contains diverse families, but it is absent in the tetraodon lineage (Fig. 6a). BEL/PAO is the second most abundant LTR group in teleost species and is represented by three distinct clades (Suzu, PAO, and Sinbad), but this group is absent in the tetraodon genome (Fig. 6b). The Suzu clade, which is homologous with the known reference elements [23], contains several families from the zebrafish, stickleback, and medaka; the PAO clade contains one family from the medaka genome and a number of families from the zebrafish, with a certain degree of structural similarity to the Zebel reference element identified in previous study [23]; while the Sinbad clade is very diverse, with three distinct branches, and contains many families from the stickleback and zebrafish genomes (Fig. 6b), which have homology to the known Kobel reference identified previously [23]. The Copia, DIRS, and Ngaro groups show very little family structure compared with the BEL/PAO and Gypsy groups (Fig. 6b).

**Table 4:** Distribution of LTR families in teleost genomes^a^

Group	Clade	Zebrafish	Medaka	Stickleback	Tentradon
Total		261	38	77	8
BEL		54	6	11	
	Suzu	2	1	2	
	Sinbad	32	4	9	
	PAO	20	1		
Copia		4	1	3	
DIRS		3			
ERV		10	1	16	
	Epsilonretrovirus	9	1	16	
	Spumaretrovirus	1			
Ngaro		5			
Gypsy		190	37	61	8
	Osvaldo/Gmr	61	3	22	1
	Barthez	60	11	10	1
	Skipper	4	1	1	1
	CsRN1		1	2	1
	V-clade	29	17	19	
	ReTe1	8	3	2	3
	Mag	28	1	5	1

^a^The newly identified LTRs by LTRHarvest and RetroTector programmes were combined with known LTRs from RepBase, and the family was built up based on the similarity of amino acid sequence of LTR elements (80 %)

In total, 10, 1, and 16 ERV families were identified in the genomes of the zebrafish, medaka, and stickleback, respectively, and no ERV families were detected in the tetraodon genome (Table 4 and Fig. 7). These ERVs were classified into 2 clades (Eplison retrovirus and Spuma retrovirus) and belong to the Class I and Class III ERV groups by phylogenetic analysis. No ERVs of Class II was detected in teleost species (Fig. 7). The majority of teleost ERVs belong to the known clade of Eplison retroviruses of Class I ERV, which has been reported in fishes and *Xenopus* [27, 28]. Only one ERV, from the zebrafish genome, is branched with known foamy virus proteins from mammals [29], and classified as the Spuma clade of Class III ERV (Fig. 7).

### Differential proliferation dynamics of class I TEs across the four teleost genomes

**Fig. 8:**
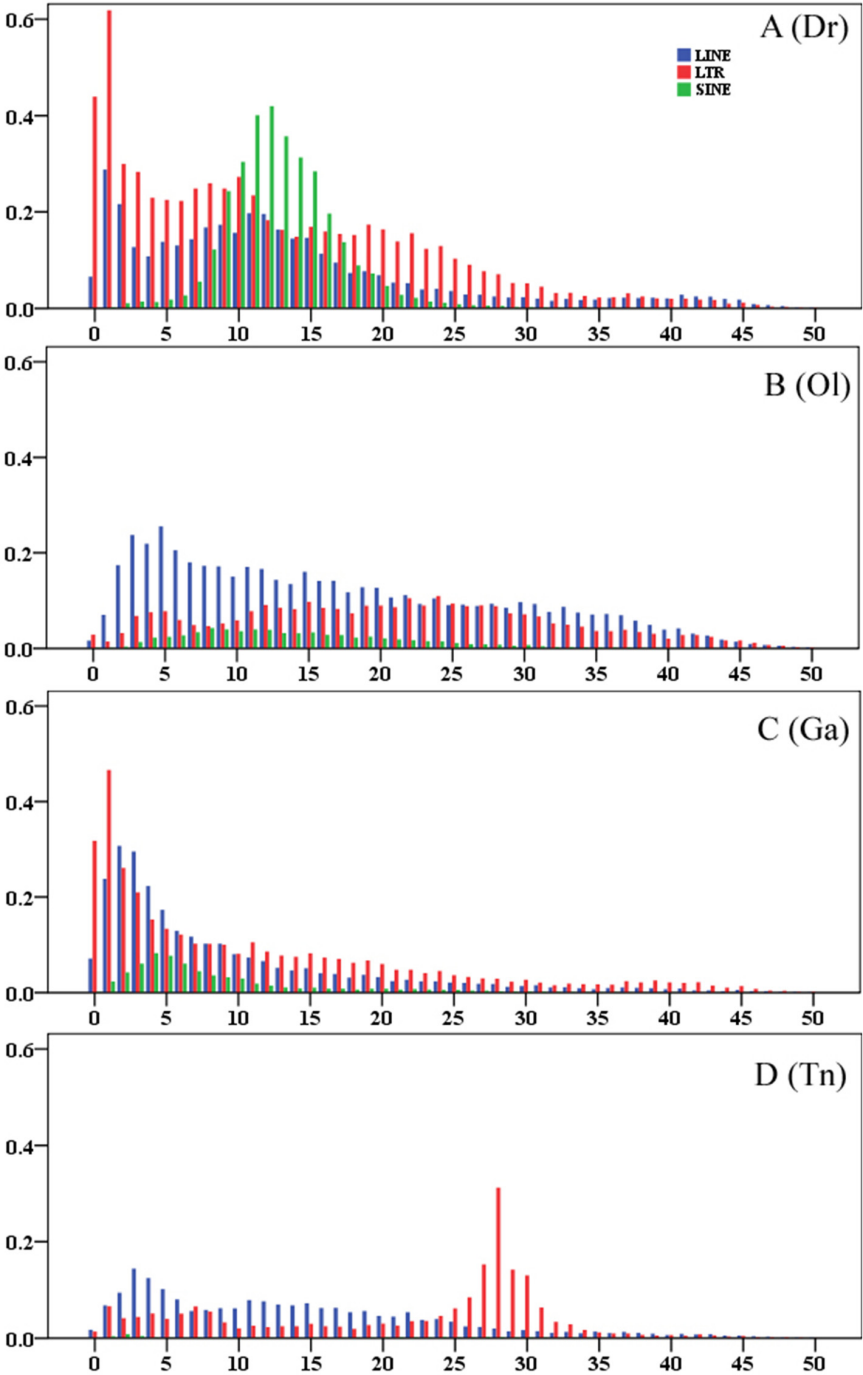
Divergence distribution of retrotransposon types (LINE, LTR, and SINE) in the zebrafish (**a**), medaka (**b**), stickleback (**c**), and tetradon (**d**) genomes. The x-axis represents the substitution rate from consensus sequences (%), and the y-axis represents the percentage of the genome comprised of repeat classes (%)

A comparison of the age and abundance distributions of TEs across the four teleost genomes revealed contrasting proliferation dynamics both between class I TEs (SINE, LINE, and LTR) and between species (Fig. 8 and Additional file 4: Table S4).

Generally, the retrotransposons within the larger genomes of the zebrafish and medaka have been active over an extended time period, in contrast with the predominantly recent activity in the smaller stickleback genome, or the extremely low level of activity in the tetraodon genome (Fig. 8). Both LTRs and LINEs in the zebrafish and stickleback genomes show evidence of very strong recent activity, in contrast to the recent decrease in activity for most types of retrotransposons in teleost species. Compared with other retrotransposons, SINEs present a very low level of activity in most teleost species, except for the zebrafish, where this repeat type has undergone one round of substantial accumulation between the divergence of 10 and 15 %, followed by a dramatic decrease in recent activity. Current activity is very limited, as shown by the distribution of very few repeats with <5 % divergence from the consensus (Fig. 8).

The clades of L1, L2, RTE, and Rex-Babar are the major repeat types of LINE in teleost species and have experienced substantial expansion during their evolutionary histories, while the other clades did not get significant amplification (Additional file 4: Table S4). The predominant clade of LINEs in most teleost genomes is L2, which contributes 1.61, 1.57, and 1.20 % to the genomes of the zebrafish, medaka, and stickleback, respectively (Additional file 4: Table S4). An in-depth divergence analysis revealed that the L2 clade has been highly active over an extended time period and shows predominantly recent activity in these teleost species (Additional file 5: Figure S1A, B, and C). The second most abundant clade of LINEs in zebrafish is L1, which represents 1.24 % coverage of the genome, with highly recent activity (Additional file 4: Table S4 and Additional file 5: Figure S1). RTE in medaka and Rex-Babar in stickleback represent the second most abundant clade of LINEs, respectively, Rex-Babar is the major clade of LINE in the tetraodon lineage, whereas the activity of all other clades of LINE within this lineage is very limited (Additional file 4: Table S4 and Additional file 5: Figure S1). The substantial recent expansion of Rex-Babar within the stickleback and tetraodon genomes was in contrast with the weak accumulation of this clade in the lineages of the zebrafish and medaka (Additional file 4: Table S4 and Additional file 5: Figure S1).

The most abundant group of LTRs in all four teleost species is Gypsy, which comprises 2.42, 1.24, 1.85, and 1.24 % of the zebrafish, medaka, stickleback, and tetraodon genomes, respectively. This group exhibits a distinct mode of evolution with a substantially recent accumulation within the zebrafish and stickleback genomes, in contrast with the relatively old proliferation dynamics within the medaka and tetraodon lineages (Additional file 4: Table S4 and Additional file 6: Figure S2). The DIRS group shows significant proliferation only in the zebrafish lineage (1.06 %) with predominantly recent activity, which is very rare within the other three teleost species. Substantial expansion of ERVs within the zebrafish (0.66 %) and stickleback (0.96) lineages was observed, which is relatively higher than that in the medaka (0.08 %) and tetraodon (0.18 %) lineages; while apparent accumulations of Ngaro in the zebrafish (0.89 %) and medaka (0.65) lineages were observed, compared to an extremely low abundance in the stickleback (0.11 %) and tetraodon (0.12 %) lineages (Additional file 4: Table S4 and Additional file 6: Figure S2).

## Discussion

### TE proliferation and genomic expansion in teleosts

Using species-specific TE libraries, which combine the update RepBase database, and the de novo repeats extracted by multipiplines, we re-annotated the mobilomes of the four representative teleosts (zebrafish, medaka, stickleback, tetraodon). The estimated fraction of repeats within zebrafish in this study (56.49 %) is similar to the 52.2 % of the previous report [16], and substantially higher than that of most investigated vertebrates, including carp (31.3 %) [30], lizards (34.4 %) [31], western clawed frog (34.5 %) [32], and birds (7–9 %) [33, 34], but comparable to the 45–52 % density in some mammalian genomes [35]. However, the coverage of repeat contents in the genome of the medaka (33.70 %) by this study is much higher (about 16.2 %) than that in the early TE annotation of the medaka genome [15]. This disagreement may be due to a significant original underestimation, since the medaka repeat database is far from complete and dense repeat regions are underrepresented in the previous draft assembly. While the density of interspersed repeats in the tetraodon genome (7.13 %) is clearly higher than the 2.7 % observed in the its close relative, fugu [4], previous size estimations suggested that the tetraodon genome might be more compact than the genome of fugu [36]. The coverage of repeats within the stickleback genome (14.21 %) annotated in the current study is far below the 25.2 % of the previous estimate [14]; the cause of this discrepancy is unclear, since the annotation method in that report is unavailable.

In this study, we confirmed that teleosts are unique among vertebrates in their overall TE composition, which represents an extraordinarily different expansion of TEs (7.13–56.49 %) across four lineages that far exceeds the variation of TEs reported in extant mammals (36–52 %) [8, 35], salamanders (25–48 %) [37], or birds (7–9 %) [33, 34]. The relationship between genome size and TE coverage in different organisms has previously revealed a general positive trend [5, 18, 38, 39]; species with larger genomes have commensurately larger proportions of TE-derived DNA. Our findings confirmed this correlation within the four teleost lineages, and the total TE contents estimated for our four teleost species match very well with the predictions based on genome size, which were well illustrated by the smallest genome of the tetraodon (7.13 % comprised of TEs) and the largest genome of the zebrafish (56.49 % comprised of TEs). Furthermore, this study uncovered that the difference is largely due to the differential expansion of class II TEs (DNA transposons) across the four teleost species. These results suggest that the differential expansion of TEs, particularly DNA transposons, is a major molecular mechanism contributing to the size variation of genomes in the four teleost species. This is similar to that in western clawed frog as an amphibian [32], but contrasts with most mammals and reptiles, where the expansion of the genome is dominated by LTR or non-LTR retrotransposons [7, 8, 10, 31, 37].

### Comparison of the diversity and activity of TEs between the four teleost genomes

In the current study, we found that teleost fish genomes represent extremely high diversity of TEs compared with the other vertebrate genomes, which is in agreement with the previous studies [18, 21, 22, 40]; furthermore, we performed a systematic comparative analysis of the intra-lineage diversity and activity of TEs across the four teleosts, and our data suggested that the differences in genome content among taxa are not limited to differences in a specific type of TE accumulation. The differences in both the diversity and activity of TEs contribute to the variances of TEs across teleost lineages. The diversity of TEs at the group level across teleost genomes is broadly similar, but the diversity at the clade (superfamily) and family level shows significant differences, and the smaller genomes have reduced clade (superfamily) and family diversity compared with the larger genomes, which has also been observed in snake lineages [41]. On the other hand, species differences in TE activity may result in changes in TE accumulation as well. In the current study, we found that zebrafish, with a fairly high TE content, represents a long-lasting and higher level of TE activity in its evolutionary history compared with the other three teleost lineages, and many DNA, LTR and LINE families show evidence of recent and ongoing proliferation, while most types of these transposons in the medaka, stickleback, and tetraodon genomes represent either a relatively young expansion and/or a rapid decrease in activity, or extremely low activity during their evolutionary history. Uncovering the reasons of the variation of diversity and activity across these teleost species is a very difficult task, particularly because TEs can also be introduced through horizontal transfer into lineages. The fertilization way, body temperature, and host defense mechanisms in opposition to TE activity (or family competition) have been suggested as biological features that may shape susceptibility to TEs in vertebrates [42, 43]. Internal fertilization may minimize exposure of gametes (and embryos) to horizontal transfer of TEs compared with external fertilization, however the four teleost lineages share the same fertilization way, and the body temperature of the four investigated teleosts, varying with the temperature of their surroundings, may also not be the principal determinant. Thus the family competition, the capacity to replicate and compete with other TEs, which is determined by the host defense mechanisms and TE itself, may be the major determinant of TE differences across the four teleost species. At least two host controlling mechanisms of the family competition of TEs: (i) cosuppression usually mediated by small interfering RNA (siRNA) and (ii) methylation, have been proved in *C. elegans* [44] and mice [45], may play roles in the evolution of diversity and activity of TEs in teleost as well. However, tests of these hypotheses and critical reevaluation will be required for further deep understanding of the regulation, mobility, and rates of expansion and extinction of TEs in teleosts.

### Evolutionary dynamics of TEs in teleost genomes compared with other vertebrates

Evolutionary dynamics of TEs between vertebrates differ drastically. The genomes of mammals and birds contain few types of TE lineages which are very abundant but relatively inactive [7, 10, 33, 34]. However, our study distinctly shows that the level of class I and class II transposon diversity and activity in teleost genomes is much higher than that seen in either bird or mammalian genomes [16, 39, 46, 47], is similar to that observed in coelacanths [48] and cod [49], and comparable with the prevalence in lizards and western clawed frog [31, 32]. Recently active TEs (with a divergence of less than 5 %) are more common in teleost genomes than in mammals or birds [8, 10, 33, 34].

The estimated fractions of LINEs in teleost genomes (1.97–4.97 %) are substantially lower than in lizards (12.34 %) and mammals (about 20 %) [6, 8, 10, 31], and comparable to that of birds (6 %), coelacanths (6.43 %), cod (3.3 %), and western clawed frog (5.4 %) [32–34, 48, 49]. However, LINEs within teleost genomes represent extremely high diversity with 6 groups. The L1 clade of LINEs contains numerous families and shows signs of recent activity. Some clades of LINEs were observed in teleost genomes, but were absent from western clawed frog, lizards, chickens and humans [10, 31, 32, 34]. Many LINE clades and families within teleost genomes seem to be recent insertions, based on their divergence analysis; this is similar to the proliferation dynamics of LINEs in lizards and western clawed frog [31, 32]. Among these is an unusually high diversity of very young families of L1 retrotransposons in the zebrafish genome, which represents the most diverse group of LINEs, containing four branches (Swimmer, Tx1-a, Tx1-b, and Tx1-c). Each branch yields highly prolific families, yet this group only covers 1.24 % of the zebrafish genome. This contrasts with observations of both mammalian and bird genomes, where only a single active family of L1 of LINEs has predominated over 10 Mya, with about a 20 % coverage of genome. In birds the most predominant TE elements are CR1 LINEs (about 6 % of the genome) and these have been demonstrated to be degenerated and nonfunctional [7, 10, 34].

Compared to lizards, western clawed frog, mammals, and birds [7, 10, 31, 32, 34], LTR retrotransposons are also very diverse and active in teleost genomes. Representatives of the seven major groups of LTR elements, including endogenous retroviruses (BEL/PAO, Copia, DIRS, ERV, Gypsy, Ngaro), with diverse clades and numerous families were identified. In particular, an unexpectedly high diversity of Gypsy (7 clades) and BEL/PAO (3 clades) were found in teleost genomes, and each clade contains diverse active families. While the Ngaro group is absent in western clawed frog and lizards [31, 32], only ERV may still be active in birds and mammals, and all other LTR groups (BEL/PAO, Copia, DIRS, Gypsy, and Ngaro) are absent or only present as fossils [7, 9, 33, 34]. This high diversity of LTR retrotransposons was already noted within teleost genomes in previous analysis [14, 40]. The estimated fractions of LTRs within the lineages vary from 1.95 % of the tetraodon genome to 5.90 % of the zebrafish, which are substantially higher than in coelacanths (0.86 %), and comparable to that in cod (4.88 %) and western clawed frog (1.75 %) [32, 48, 49].

Teleosts are unique among vertebrates in their proliferation dynamics of DNA transposons; DNA transposons vary dramatically in abundance across teleost species, dominate the variations in genome size, and also represent the highest level of diversity among vertebrates. The coverage of DNA transposons varies across teleost genomes, from 1.55 % in the tetraodon genome to 41.07 % in the zebrafish. The zebrafish genome contains a marked excess of DNA transposons, which is unique among sequenced vertebrate genomes, and is substantially higher than in very close lineages of carp (17.53 %). Indeed, only western clawed frog genome, which is comprised of 25 % DNA transposons, are comparable. The estimated fractions of DNA transposons in the medaka (11.00 %) and stickleback (4.47 %) genomes are substantially higher than in coelacanths (0.20 %) [48], lungfish (1.3 %) [50], birds (less than 1 %) [33, 34] and mammals (less than 3 %) [7, 10], but comparable to that in lizards (8.86 %) [31], salamanders (6.37 %) [37], and cod (6.39 %) [49].

The diversity of teleost DNA transposons, which was already noted previously [18, 30], far exceeds that in other examined vertebrates, including mammals, birds, coelacanths, cod, lizards, and western clawed frog [31, 32, 34, 46, 48]. A particularly high abundance and diversity of hAT and Tc1/Mariner was found in teleost genomes. Nine superfamilies of DNA transposons, including Ginger, Sola, CMC-EnSpm, Crypton, Dada, MULE-MuDR, P, PIF-ISL2EU, and Academ, were observed in teleosts that were absent in lizards, western clawed frog, and coelacanths [31, 32, 48]. In addition, diverse autonomous hAT and Tc1/Mariner subfamilies were identified in teleost genomes, suggesting that the DNA transposons seem to be relatively young and active in teleosts, in contrast to the few recently active DNA transposons found in mammals and birds [7, 10, 33, 34]. Overall, teleosts have an extremely wide diversity and high level of activity of TEs, but represent a significantly different success of TEs across lineages, while mammalian genomes are enriched with L1 elements but a low level of diversity and have a high degree of TE expansion, and bird genomes exhibit low TE density with very little mobile element activity.

## Conclusion

In this study, we investigated the diversity, activity, and abundance distribution of TEs among four closely related teleost species. In contrast to other vertebrates, teleosts display contrasting profiles of mobilomes across the four investigated lineages. The larger genomes represent a higher diversity and activity within each family and a greater abundance of TEs compared with the smaller genomes. The differences in TE expansion, dominated by DNA transposons, explain the main size variation in the four teleost genomes, and the species differences in both the diversity and activity of TEs contribute to the variations in TE accumulations. TEs play pivotal roles in teleost genome evolution.

## Methods

### Computational identification of interspersed repeats

The zebrafish (GRCz10), medaka (MEDAKA1), stickleback (BROADS1), and tetraodon (TETRAODON8) genomes were downloaded from the Ensembl database (http://asia.ensembl.org/index.html). The repeat contents of the zebrafish, medaka, stickleback and tetraodon genomes were assessed using RepeatMasker (http://www.repeatmasker.org/), RepeatModeler (http://repeatmasker.org/RepeatModeler.html) and ab initio repeat prediction programmes. The RepBase (http://www.girinst.org/) of consensus repeat sequences [51] was used to identify repeats in the genome derived from known classes of elements. RepeatModeler was used to build de novo repeats. The autonomous hAT and Tc1/Mariner DNA transposons were queried using TBLASTN to detect the presence of coding sequences related to all known DNA transposon superfamilies in RepBase [51]. The top 10–40 non-overlapping hits (generally Evalue <10^−5^) were extracted, along with 500 bp of flanking sequence, aligned using a local installation of MUSCLE [52], and used to construct consensus sequences. For each consensus, coding sequences were predicted by using Open Reading Frame (ORF) Finder (http://www.ncbi.nlm.nih.gov/projects/gorf/). The non-LTR retrotransposons were identified by MGEScan-non-LTR [19], and the LTR retrotransposons, including endogenous retroviruses (ERVs), were identified by LTRharvest [47] and RetroTector [53]. The autonomous LTRs were classified into families based on amino acid sequence similarity (80 %) of the ORF containing RT domain; while the autonomous LINEs were classified into families based on the structure of ORFs and amino acid sequence similarity (80 %) of the ORF2.

Repeats characterized as putative TEs by the previous approach were joined to the RepBase database of TEs (update 20150807), and the redundancies were filtered out to create a custom library for comparison to find the distribution and coverage of TEs in the genome using RepeatMasker (RepeatMasker -open-4.0.5). The redundant repeats were removed based on the 80-80 rule, which considers two sequences as belonging to same TE family if they can be aligned over more than 80 % of their length, with over 80 % identity. The new non-redundant repeats of the four teleost species were given in fasta file format in Additional files (Additional files 7, 8, 9 and 10).

### Phylogenetic analysis

Bootstrapped (1000 replicates) neighbour-joining (NJ) phylogenetic trees were generated using MEGA5 [54] based on a muscle multiple protein alignment with the conserved domain of the DNA transposases or RT (reverse transcription) domain of retrotransposons. For the hAT superfamily, we used a conserved 39 aa-long region of hAT transposase [55] to build the alignment, and then deduced the NJ tree. For the Tc1/Mariner superfamily, the NJ tree was generated by using a multiple sequence alignment with the most conserved domain of the Tc1/Mariner transposase (about 150 aa) corresponding to the catalytic “DDE” domain, as in [56]. For retrotransposons (LINEs, LTRs and ERVs), the NJ tree was generated by using an amino acid multiple alignment of the conserved RT domain from retrotransposons and reference elements. All these alignment are available upon request.

### Divergence distribution of interspersed repeats

The average number of substitutions per site (K) for each fragment was estimated according to the divergence levels reported by RepeatMasker, using the one-parameter Jukes-Cantor formula K = −300/4 × Ln(1–D × 4/300) as in [7], where D represents the proportion of sites that differ between the fragmented repeat and the consensus sequence.

## Additional files

Additional file 1: Table S1. Numbers of families of DNA repeats from RepBase and the de novo repeats identified by TBLASTN. (PDF 91 kb) Additional file 2: Table S2. Characteristics of Non-LTR retrotransposons present in teleost genomes, separated by family. (PDF 158 kb) Additional file 3: Table S3. Characteristics of LTR retrotransposons present in teleost genomes, separated by family. (PDF 371 kb) Additional file 4: Table S4. Abundance of retrotransposons in teleost genomes. (PDF 181 kb) Additional file 5: Figure S1. Divergence distribution of the major clades of LINEs in the zebrafish (A), medaka (B), stickleback (C), and tetraodon (D) genomes. The x-axis represents the substitution rate from consensus sequences (%), and the y-axis represents the percentage of the genome comprised of repeat classes (%). (JPG 285 kb) Additional file 6: Figure S2. Divergence distribution of the major groups of LTRs in the zebrafish (A), medaka (B), stickleback (C), and tetraodon (D) genomes. The x-axis represents the substitution rate from consensus sequences (%), and the y-axis represents the percentage of the genome comprised of repeat classes (%). (JPG 312 kb) Additional file 7:
**TE sequence 1: new repeats identified in zebrafish.** (FAS 390 kb) Additional file 8:
**TE sequence 2: new repeats identified in medaka.** (FAS 376 kb) Additional file 9:
**TE sequence 3: new repeats identified in stickleback.** (FAS 357 kb) Additional file 10:
**TE sequence 4: new repeats identified in tetraodon.** (FAS 97 kb)

## Abbreviations

Dr: danio rerio
ERV: endogenous retroviruse
Ga: gasterosteus aculeatus
LINE: long interspersed nuclear elements
LTR: long terminal repeats
Mb: mega base pairs
NJ: neighbour-joining
ORF: open reading frame
Ol: oryzias latipes
RT: reverse transcription
SINE: short interspersed nuclear elements
TSD: target site duplication
TIR: terminal inverted repeat
Tn: Tetraodon nigroviridis
TEs: Transposable elements
