# Supplementary material for: The contribution of transposable elements to size variations between four teleost genomes
Source: Mob DNA. 2016 Feb 9;7:4. doi: 10.1186/s13100-016-0059-7 (PMC4746887; doi:10.1186/s13100-016-0059-7)
Supplement: Additional file 1: Table S1. — Numbers of families of DNA repeats from RepBase and the de novo repeats identified by TBLASTN. (PDF 91 kb) [file 13100_2016_59_MOESM1_ESM.pdf]

Additional File 1: Table S1. Numbers of families of DNA repeats from RepBase and the de novo repeats identified by TBLASTN*.

Superfamily	Zebrafish				Medaka				Stickleback				Tetraodon			
	RBF	RMF	TBF	NRF	RBF	RMF	TBF	NRF	RBF	RMF	TBF	NRF	RBF	RMF	TBF	NRF
Total	768	628		1249	18	202		234	12	139		161	1	67		74
Academ	1			1		1		1	1	1		2				
CMC-Chapaev-3					1			1								
CMC-EnSpm	75	54		120		1		1		9		9		3		3
Crypton	8	1		9	3	3		5								
Dada	6			6	2			2	1			1	1			1
Ginger	4	1		5		1		1								
Kolobok	38	29		60	1			1		2		2				
Merlin	4			4						2		2				
MULE-MuDR	3	1		3		1		1		1		1				
PIF-Harbinger	45	29		58		14		14	2	7		9		2		2
PIF-ISL2EU	5	2		6		1		1		1		1				
IS3EU	10			10												
P	5	3		7												
PiggyBac	16	19		32	1	6		6		3		3		2		2
Sola	2	7		7		1		1		1		1				
Zisupton	8			8												
Tc1/Mariner (total)	65	77		130	8	54		71	5	27		36		24		25
ISRm11	8	4		11	3	1		4	5	4		8		2		2
Stowaway	2	1		3						1		1				
Tc1	37	44	4	71	5	37	11	49		17	5	20		11	4	11

pogo	4	4	1	9		16	3	18		5	2	7		11	1	12
Unclassified Tc1/Mariner	14	24		36												
hAT (total)	299	247		498	2	105		114	3	67		76		34		39
Ac	117	88		191	1	34	2	37	1	20	3	25		5		7
Blackjack	1			1		1		1						1		
Charlie	63	43		98		44	4	48	1	21	3	25		16	5	20
Tag1										1		1				
Tip100	25	17		40		9	1	10		10	1	10		5		5
Tol2	5	1		6		1		1		1		1		3		3
hAT5	13	7		18		2		2	1	2		3				
hAT6	2	1		3												
hATx						1		1								
hobo	2	2		2												
Unclassified hAT	71	88		139	1	13		14		12		11		4		4
Unclassified DNA TEs	170	157		280		14		14		16		16		1		1
	4	1		5						2		2		1		1
Self-synthesizing TE																
Maverick	4	1		5						2		2		1		1
Rolling-circle TE																
Helitron	13	21		33		1		1		3		3		1		1
Number of total superfamily		18				14				13				7		

*RBF, RepBase families; RMF, RepeatModeler families; TBF, TBLASTN families; NRF, Non-Redundant families, the Non-Redundant families combing families from RepBase database, RepeatModeler, and TBLASTN; The redundant repeats were removed based on the 80-80 rule, which considers two sequences as belonging to same TE family if they can be aligned over more than 80% of their length, with over 80%

identity.
