# Supplementary material for: The contribution of transposable elements to size variations between four teleost genomes
Source: Mob DNA. 2016 Feb 9;7:4. doi: 10.1186/s13100-016-0059-7 (PMC4746887; doi:10.1186/s13100-016-0059-7)
Supplement: Additional file 2: Table S2. — Characteristics of Non-LTR retrotransposons present in teleost genomes, separated by family. (PDF 158 kb) [file 13100_2016_59_MOESM2_ESM.pdf]

Additional file 2: Table S2. Characteristics of Non-LTR retrotransposons present in teleost genomes, separated by family.

Species	Clade	Family	Branch	RepBase family	FL Size (bp)	Copy number	FL Copy Number	Nb. AAs ORF1	Nb. AAs ORF2
Zebrafish	L1	L1-Dr1	swimmer	L1-1_Dr	6606	11	3	294	1277
Zebrafish	L1	L1-Dr2	swimmer	L1-10_Dr	6594	9	3	281	1260
Zebrafish	L1	L1-Dr3	swimmer	L1-104_Dr	6609	4	1	272	1273
Zebrafish	L1	L1-Dr4	swimmer	L1-11_Dr	6606	16	2	264	1244
Zebrafish	L1	L1-Dr5	swimmer	L1-111_Dr					
Zebrafish	L1	L1-Dr6	swimmer	L1-114	6597	6	1	217	1125
Zebrafish	L1	L1-Dr7	swimmer	L1-12_Dr	6606	5	2	352	1261
Zebrafish	L1	L1-Dr8	swimmer	L1-120	6615	10	3	330	1263
Zebrafish	L1	L1-Dr9	swimmer	L1-13_Dr	6606	5	1	295	1270
Zebrafish	L1	L1-Dr10	swimmer	L1-14_Dr					
Zebrafish	L1	L1-Dr11	swimmer	L1-16_Dr					
Zebrafish	L1	L1-Dr12	swimmer	L1-17_Dr					
Zebrafish	L1	L1-Dr13	swimmer	L1-18_Dr					
Zebrafish	L1	L1-Dr14	swimmer	L1-19_Dr	6600	5	1	292	1309
Zebrafish	L1	L1-Dr15	swimmer	L1-190	6600	4	2	300	1269
Zebrafish	L1	L1-Dr16	swimmer	L1-20_Dr	6600	4	1	265	1274
Zebrafish	L1	L1-Dr17	swimmer	L1-21_Dr	6609	7	1	334	1265
Zebrafish	L1	L1-Dr18	swimmer	L1-22_Dr	6609	11	1	328	1265
Zebrafish	L1	L1-Dr19	swimmer	L1-23_Dr	6612	9	3	295	1259
Zebrafish	L1	L1-Dr20	swimmer	L1-24_Dr	6606	7	4	358	1270
Zebrafish	L1	L1-Dr21	swimmer	L1-25_Dr	6615	7	2	329	1266
Zebrafish	L1	L1-Dr22	swimmer	L1-30_Dr	6597	7	1	278	1219
Zebrafish	L1	L1-Dr23	swimmer	L1-31_Dr	6600	14	1	344	1262
Zebrafish	L1	L1-Dr24	swimmer	L1-32_Dr	6603	15	5	287	1272
Zebrafish	L1	L1-Dr25	swimmer	L1-33_Dr	6600	5	4	287	1264
Zebrafish	L1	L1-Dr26	swimmer	L1-34_Dr	6600	9	5	268	1270
Zebrafish	L1	L1-Dr27	swimmer	L1-35_Dr	6600	10	3	301	1078
Zebrafish	L1	L1-Dr28	swimmer	L1-38_Dr	6594	7	3	279	1242
Zebrafish	L1	L1-Dr29	swimmer	L1-40_Dr	6606	7	2	364	1260
Zebrafish	L1	L1-Dr30	swimmer	L1-42_Dr	6603	7	2	283	1274

Zebrafish	L1	L1-Dr31	swimmer	L1-44_Dr					
Zebrafish	L1	L1-Dr32	swimmer	L1-48_Dr	6591	3	1	293	1255
Zebrafish	L1	L1-Dr33	swimmer	L1-49_Dr					
Zebrafish	L1	L1-Dr34	swimmer	L1-51_Dr					
Zebrafish	L1	L1-Dr35	swimmer	L1-55_Dr	6603	6	2	280	1260
Zebrafish	L1	L1-Dr36	swimmer	L1-6_Dr	6600	9	6	306	1255
Zebrafish	L1	L1-Dr37	swimmer	L1-66_Dr	6600	7	2	273	1258
Zebrafish	L1	L1-Dr38	swimmer	L1-67_Dr	6612	5	1	230	1264
Zebrafish	L1	L1-Dr39	swimmer	L1-68_Dr					
Zebrafish	L1	L1-Dr40	swimmer	L1-69_Dr	6600	9	3	275	1273
Zebrafish	L1	L1-Dr41	swimmer	L1-72_Dr	6606	3	2	277	1264
Zebrafish	L1	L1-Dr42	swimmer	L1-8_Dr	6603	9	4	290	1260
Zebrafish	L1	L1-Dr43	swimmer	L1-82_Dr	6603	5	1	347	1262
Zebrafish	L1	L1-Dr44	swimmer	L1-84_Dr	6606	6	5	298	1268
Zebrafish	L1	L1-Dr45	swimmer	L1-94_Dr	6603	5	4	-	1271
Zebrafish	L1	L1-Dr46	Tx1-a	L1-74_Dr					
Zebrafish	L1	L1-Dr47	Tx1-a	Tx1-11_Dr	6585	7	2	320	1244
Zebrafish	L1	L1-Dr48	Tx1-a	Tx1-12_Dr	6591	8	1	282	1240
Zebrafish	L1	L1-Dr49	Tx1-a	Tx1-18_Dr					
Zebrafish	L1	L1-Dr50	Tx1-a	Tx1-19_Dr					
Zebrafish	L1	L1-Dr51	Tx1-a	Tx1-2_Dr	6588	6	2	292	1239
Zebrafish	L1	L1-Dr52	Tx1-a	Tx1-20_Dr	6588	11	3	309	1246
Zebrafish	L1	L1-Dr53	Tx1-a	Tx1-29_Dr					
Zebrafish	L1	L1-Dr54	Tx1-a	Tx1-3_Dr	6585	4	4	295	1245
Zebrafish	L1	L1-Dr55	Tx1-a	Tx1-4_Dr	6576	13	6	313	1245
Zebrafish	L1	L1-Dr56	Tx1-a	Tx1-5_Dr	6579	8	3	290	1243
Zebrafish	L1	L1-Dr57	Tx1-a	Tx1-6_Dr					
Zebrafish	L1	L1-Dr58	Tx1-a	Tx1-7_Dr	6588	9	1	282	1243
Zebrafish	L1	L1-Dr59	Tx1-a	Tx1-8_Dr	6585	4	1	266	1245
Zebrafish	L1	L1-Dr60	Tx1-b	KibiDr1					
Zebrafish	L1	L1-Dr61	Tx1-b	KibiDr2					
Zebrafish	L1	L1-Dr62	Tx1-b	L1-15_Dr	6591	14	4	419	1330
Zebrafish	L1	L1-Dr63	Tx1-b		6600	7	2	414	1323
Zebrafish	L1	L1-Dr64	Tx1-b		6594	5	1	410	1325

Zebrafish	L1	L1-Dr65	Tx1-b		6606	4	2	390	1312
Zebrafish	L1	L1-Dr66	Tx1-b		6609	5	1	395	1299
Zebrafish	L1	L1-Dr67	Tx1-b		6795	4	3	443	1322
Zebrafish	L1	L1-Dr68	Tx1-b	Tx1-1_Dr	6591	10	2	434	1310
Zebrafish	L1	L1-Dr69	Tx1-b	Tx1-10_Dr	6585	15	8	431	1333
Zebrafish	L1	L1-Dr70	Tx1-b	Tx1-25_Dr	6597	7	2	397	1323
Zebrafish	L1	L1-Dr71	Tx1-b	Tx1-26_Dr	6597	4	2	414	1323
Zebrafish	L1	L1-Dr72	Tx1-b	Tx1-32_Dr	6594	8	3	424	1318
Zebrafish	L1	L1-Dr73	Tx1-b	Tx1-33_Dr	6591	10	2	392	1311
Zebrafish	L1	L1-Dr74	Tx1-b	Tx1-34_Dr					
Zebrafish	L1	L1-Dr75	Tx1-c	KenoDr1	6561	21	3	436	1148
Zebrafish	L1	L1-Dr76	Tx1-c	L1-2_Dr					
Zebrafish	L1	L1-Dr77	Tx1-c	L1-202	6567	9	6	439	1284
Zebrafish	L1	L1-Dr78	Tx1-c	L1-3_Dr	6567	6	3	450	1279
Zebrafish	L1	L1-Dr79	Tx1-c	L1-4B_Dr	6573	19	4	485	1273
Zebrafish	L1	L1-Dr80	Tx1-c	L1-5_Dr	6570	10	2	482	1286
Zebrafish	L1	L1-Dr81	Tx1-c	Tx1-47_Dr	6558	6	1	379	1277
Zebrafish	L1	L1-Dr82	Tx1-c	Tx1-48_Dr	6564	9	5	436	1282
Zebrafish	L2	L2-Dr1		L2-2_DR	6528	140	85	-	965
Zebrafish	L2	L2-Dr2		L2-1_DR	6555	7	3	-	1130
Zebrafish	L2	L2-Dr3		L2-17_DR	6546	5	2	-	1119
Zebrafish	L2	L2-Dr4		L2-34_DR	6540	5	1	-	1131
Zebrafish	L2	L2-Dr5		L2-3_DR	6561	46	16	494	908
Zebrafish	L2	L2-Dr6		L2-3_DR	6561	26	21	516	908
Zebrafish	L2	L2-Dr7		L2-8_DR	6561	8	3	-	1054
Zebrafish	L2	L2-Dr8		L2-4_DR	6552	11	3	-	1052
Zebrafish	L2	L2-Dr9		L2-1_DR	6606	123	33	302	872
Zebrafish	L2	L2-Dr10		L2-42_Dr					
Zebrafish	L2	L2-Dr11		L2-16_Dr					
Zebrafish	I	I-Dr1		I-3_DR	6579	14	4	406	1175
Zebrafish	I	I-Dr2		Nimb-3_DR	6606	9	3	398	1258
Zebrafish	I	I-Dr3		I-1_DR	6597	12	3	417	1250
Zebrafish	I	I-Dr4		Nimb-2_DR	6609	4	3	413	1253
Zebrafish	I	I-Dr5		Nimb-8_DR	6606	5	4	395	1260

Zebrafish	I	I-Dr6		Nimb-5_Dr					
Zebrafish	I	I-Dr7		Nimb-6_Dr					
Zebrafish	I	I-Dr8		Nimb-17_Dr					
Zebrafish	I	I-Dr9		Nimb-12_Dr					
Zebrafish	R2	R2-Dr1		Togen-1_DR	5292	5	1	-	1113
Zebrafish	R2	R2-Dr2		R2Dr					
Zebrafish	REX	REX-Dr1			6624	6	1	323	1029
Zebrafish	REX	REX-Dr2		Rex1-38_DR	6477	5	3	-	964
Zebrafish	REX	REX-Dr3			6453	4	1	-	980
Zebrafish	REX	REX-Dr4		Rex1-28_DR					
Zebrafish	REX	REX-Dr5		Rex1-29_DR					
Zebrafish	REX	REX-Dr6		Rex1-40_DR					
Zebrafish	REX	REX-Dr7		Rex1-26_DR					
Zebrafish	REX	REX-Dr8		Rex1-1_DR					
Zebrafish	REX	REX-Dr9		Rex1-31_DR					
Zebrafish	RTE	RTE-Dr1		RTEX-1_DR	6831	6	2	568	1152
Zebrafish	RTE	RTE-Dr2		RTE-1_DR	6756	8	3	-	1049
Zebrafish	RTE	RTE-Dr3		RTEX-5_Dr					
Zebrafish	RTE	RTE-Dr4		RTEX-4_Dr					
Zebrafish	RTE	RTE-Dr5		EXPANDER1_Dr					
Medaka	L1	L1-Ol1	Tx1-c		6561	4	1	265	1252
Medaka	L1	L1-Ol2	Tx1-b		6597	6	1	424	1319
Medaka	L1	L1-Ol3	Tx1-c		6561	5	1	251	1022
Medaka	L1	L1-Ol4	Swimmer		6615	16	1	343	997
Medaka	L1	L1-Ol5	swimmer			8	1	-	705
Medaka	L1	L1-Ol6	swimmer		6594	21	1	-	1072
Medaka	L2	L2-Ol1			6148	15	1	-	715
Medaka	R2	R2-Ol1			5262	25	1	-	743
Stickleback	L1	L1-Ga1	Tx1-b		6597	6	1	-	1321
Stickleback	L1	L1-Ga2	Tx1-c		6546	10	1	478	1122
Stickleback	L1	L1-Ga3	Tx1-b		6594	3	1	-	1355
Stickleback	L2	L2-Ga1			6624	42	2	294	1002
Stickleback	L2	L2-Ga2			6540	40	4	-	986

Stickleback	L2	L2-Ga3		6540	15	1	-	1128
Stickleback	R2	R2-Ga1		5274	4	1	-	1234
Stickleback	REX	REX-Ga1		6543	5	1		998
Stickleback	REX	REX-Ga2						
Stickleback	RTE	RTE-Ga1	RTE-1_GA	6681	32	11	-	1060
Stickleback	RTE	RTE-Ga2		6681	24	7	-	968
Tetraodon	L1	L1-Tn1	KoshiTn1					
Tetraodon	L1	L1-Tn2	KibiTn1					
