# Supplementary material for: The contribution of transposable elements to size variations between four teleost genomes
Source: Mob DNA. 2016 Feb 9;7:4. doi: 10.1186/s13100-016-0059-7 (PMC4746887; doi:10.1186/s13100-016-0059-7)
Supplement: Additional file 3: Table S3. — Characteristics of LTR retrotransposons present in teleost genomes, separated by family. (PDF 371 kb) [file 13100_2016_59_MOESM3_ESM.pdf]

Additional file 3: Table S3. Characteristics of LTR retrotransposons present in teleost genomes, separated by family.

Species	Group	Clade	Family	RepBase family	Number of elements identified	
					LTRHarvest	RetroTector
Zebrafish	BEL/PAO	Suzu	Bel-Dr1	BELPADRE	6	2
Zebrafish	BEL/PAO	Suzu	Bel-Dr2	BEL4	5	1
Zebrafish	BEL/PAO	Sinbad	Bel-Dr3	BEL-44	1	
Zebrafish	BEL/PAO	Sinbad	Bel-Dr4	BEL-56	5	7
Zebrafish	BEL/PAO	Sinbad	Bel-Dr5	BEL65		
Zebrafish	BEL/PAO	Sinbad	Bel-Dr6	BEL1	1	1
Zebrafish	BEL/PAO	Sinbad	Bel-Dr7	BEL12	3	3
Zebrafish	BEL/PAO	Sinbad	Bel-Dr8	BEL24	8	5
Zebrafish	BEL/PAO	Sinbad	Bel-Dr9	BEL33		
Zebrafish	BEL/PAO	Sinbad	Bel-Dr10	BEL22	4	
Zebrafish	BEL/PAO	Sinbad	Bel-Dr11	BEL27	10	4
Zebrafish	BEL/PAO	Sinbad	Bel-Dr12	BEL11	3	2
Zebrafish	BEL/PAO	Sinbad	Bel-Dr13		12	2
Zebrafish	BEL/PAO	Sinbad	Bel-Dr14	BEL36	5	5
Zebrafish	BEL/PAO	Sinbad	Bel-Dr15		2	1
Zebrafish	BEL/PAO	Sinbad	Bel-Dr16		5	3
Zebrafish	BEL/PAO	Sinbad	Bel-Dr17	BEL-47	2	4
Zebrafish	BEL/PAO	Sinbad	Bel-Dr18	BEL32	2	5
Zebrafish	BEL/PAO	Sinbad	Bel-Dr19	BEL-42		1
Zebrafish	BEL/PAO	Sinbad	Bel-Dr20		1	2
Zebrafish	BEL/PAO	Sinbad	Bel-Dr21	BEL-45	1	2
Zebrafish	BEL/PAO	Sinbad	Bel-Dr22	BEL-40	2	2
Zebrafish	BEL/PAO	Sinbad	Bel-Dr23	BEL-46		
Zebrafish	BEL/PAO	Sinbad	Bel-Dr24	BEL-63		1
Zebrafish	BEL/PAO	Sinbad	Bel-Dr25	BEL35		
Zebrafish	BEL/PAO	Sinbad	Bel-Dr26	BEL5		
Zebrafish	BEL/PAO	Sinbad	Bel-Dr27	BEL31	2	1

Zebrafish	BEL/PAO	Sinbad	Bel-Dr28	BEL-58	1	2
Zebrafish	BEL/PAO	Sinbad	Bel-Dr29	BEL-39		
Zebrafish	BEL/PAO	Sinbad	Bel-Dr30	BEL-41		
Zebrafish	BEL/PAO	Sinbad	Bel-Dr31	BEL29	1	
Zebrafish	BEL/PAO	Sinbad	Bel-Dr32	BEL30	1	1
Zebrafish	BEL/PAO	Sinbad	Bel-Dr33	BEL34		1
Zebrafish	BEL/PAO	Sinbad	Bel-Dr34	BEL8	1	2
Zebrafish	BEL/PAO	PAO	Bel-Dr35	BEL-16	1	
Zebrafish	BEL/PAO	PAO	Bel-Dr36	BEL-52	2	3
Zebrafish	BEL/PAO	PAO	Bel-Dr37	BEL-54	4	2
Zebrafish	BEL/PAO	PAO	Bel-Dr38	BEL17	2	3
Zebrafish	BEL/PAO	PAO	Bel-Dr39	BEL21	2	
Zebrafish	BEL/PAO	PAO	Bel-Dr40	BEL9	1	1
Zebrafish	BEL/PAO	PAO	Bel-Dr41	BEL-26	5	8
Zebrafish	BEL/PAO	PAO	Bel-Dr42	BEL-59	7	5
Zebrafish	BEL/PAO	PAO	Bel-Dr43	BEL-62	5	4
Zebrafish	BEL/PAO	PAO	Bel-Dr44	BEL20	3	5
Zebrafish	BEL/PAO	PAO	Bel-Dr45	BEL25	2	1
Zebrafish	BEL/PAO	PAO	Bel-Dr46	BEL7	3	2
Zebrafish	BEL/PAO	PAO	Bel-Dr47			
Zebrafish	BEL/PAO	PAO	Bel-Dr48	BEL23		
Zebrafish	BEL/PAO	PAO	Bel-Dr49		1	1
Zebrafish	BEL/PAO	PAO	Bel-Dr50	BEL15	2	2
Zebrafish	BEL/PAO	PAO	Bel-Dr51		1	1
Zebrafish	BEL/PAO	PAO	Bel-Dr52	BEL-49		
Zebrafish	BEL/PAO	PAO	Bel-Dr53	BEL-57	1	
Zebrafish	BEL/PAO	PAO	Bel-Dr54	BEL2	1	2
Zebrafish	Copia		Co-Dr1	Copia1	8	8
Zebrafish	Copia		Co-Dr2		1	1
Zebrafish	Copia		Co-Dr3	Copia4	2	
Zebrafish	Copia		Co-Dr4	Copia5		

Zebrafish	DIRS		Di-Dr1	Dirs1	5	28
Zebrafish	DIRS		Di-Dr2	DIRS-13		
Zebrafish	DIRS		Di-Dr3	DIRS-3		
Zebrafish						
Zebrafish	ERV	Epsilon	ERV-Dr1		1	1
Zebrafish	ERV	Epsilon	ERV-Dr2		2	6
Zebrafish	ERV	Epsilon	ERV-Dr3	ERV1-1	1	8
Zebrafish	ERV	Epsilon	ERV-Dr4	ERV1		4
Zebrafish	ERV	Epsilon	ERV-Dr5	ZFERV		3
Zebrafish	ERV	Epsilon	ERV-Dr6	ERV4	1	2
Zebrafish	ERV	Epsilon	ERV-Dr7	ERV3		
Zebrafish	ERV	Epsilon	ERV-Dr8			2
Zebrafish	ERV	Epsilon	ERV-Dr9	ZFERV-2	1	1
Zebrafish	ERV	Spuma	ERV-Dr10	ERV1-2		2
Zebrafish	Ngaro		Ng-Dr1	Dirs-6B		
Zebrafish	Ngaro		Ng-Dr2	DIRS-9C		1
Zebrafish	Ngaro		Ng-Dr3	DIRS-16		
Zebrafish	Ngaro		Ng-Dr4	DIRS-15		
Zebrafish	Ngaro		Ng-Dr5	DIRS-4		
Zebrafish	Gypsy	Barthez	Gy-Dr1		1	
Zebrafish	Gypsy	Barthez	Gy-Dr2			1
Zebrafish	Gypsy	Barthez	Gy-Dr3	GYPSY-126	1	3
Zebrafish	Gypsy	Barthez	Gy-Dr4	GYPSY-16		16
Zebrafish	Gypsy	Barthez	Gy-Dr5	GYPSY147	1	3
Zebrafish	Gypsy	Barthez	Gy-Dr6			1
Zebrafish	Gypsy	Barthez	Gy-Dr7	GYPSY71		2
Zebrafish	Gypsy	Barthez	Gy-Dr8	GYPSY-21		5
Zebrafish	Gypsy	Barthez	Gy-Dr9		1	1
Zebrafish	Gypsy	Barthez	Gy-Dr10	GYPSY102	1	6
Zebrafish	Gypsy	Barthez	Gy-Dr11	GYPSY83		3
Zebrafish	Gypsy	Barthez	Gy-Dr12	GYPSY51	1	3

Zebrafish	Gypsy	Barthez	Gy-Dr13	GYPSY-233		1
Zebrafish	Gypsy	Barthez	Gy-Dr14	GYPSY100		6
Zebrafish	Gypsy	Barthez	Gy-Dr15			1
Zebrafish	Gypsy	Barthez	Gy-Dr16	GYPSY84		4
Zebrafish	Gypsy	Barthez	Gy-Dr17	GYPSY132	2	4
Zebrafish	Gypsy	Barthez	Gy-Dr18	GYPSY82		7
Zebrafish	Gypsy	Barthez	Gy-Dr19	GYPSY-162		2
Zebrafish	Gypsy	Barthez	Gy-Dr20	GYPSY11		6
Zebrafish	Gypsy	Barthez	Gy-Dr21		3	2
Zebrafish	Gypsy	Barthez	Gy-Dr22			1
Zebrafish	Gypsy	Barthez	Gy-Dr23			1
Zebrafish	Gypsy	Barthez	Gy-Dr24	GYPSY-15		10
Zebrafish	Gypsy	Barthez	Gy-Dr25	GYPSY80		3
Zebrafish	Gypsy	Barthez	Gy-Dr26	GYPSYG-198		3
Zebrafish	Gypsy	Barthez	Gy-Dr27	GYPSY-14		4
Zebrafish	Gypsy	Barthez	Gy-Dr28	GYPSY98	1	4
Zebrafish	Gypsy	Barthez	Gy-Dr29			1
Zebrafish	Gypsy	Barthez	Gy-Dr30	GYPSY10	1	9
Zebrafish	Gypsy	Barthez	Gy-Dr31	GYPSY116		7
Zebrafish	Gypsy	Barthez	Gy-Dr32	GYPSY117		5
Zebrafish	Gypsy	Barthez	Gy-Dr33	GYPSY118		9
Zebrafish	Gypsy	Barthez	Gy-Dr34	GYPSY12		3
Zebrafish	Gypsy	Barthez	Gy-Dr35	GYPSY13		6
Zebrafish	Gypsy	Barthez	Gy-Dr36	GYPSY133		2
Zebrafish	Gypsy	Barthez	Gy-Dr37	GYPSY134	1	
Zebrafish	Gypsy	Barthez	Gy-Dr38	GYPSY136	1	3
Zebrafish	Gypsy	Barthez	Gy-Dr39	Gypsy137	2	3
Zebrafish	Gypsy	Barthez	Gy-Dr40	GYPSY140		
Zebrafish	Gypsy	Barthez	Gy-Dr41	GYPSY144		2
Zebrafish	Gypsy	Barthez	Gy-Dr42	GYPSY156		2
Zebrafish	Gypsy	Barthez	Gy-Dr43	GYPSY157		2
Zebrafish	Gypsy	Barthez	Gy-Dr44	GYPSY-17		2
Zebrafish	Gypsy	Barthez	Gy-Dr45	GYPSY-18		2

Zebrafish	Gypsy	Barthez	Gy-Dr46	GYPSY-19		2
Zebrafish	Gypsy	Barthez	Gy-Dr47	GYPSY-196		1
Zebrafish	Gypsy	Barthez	Gy-Dr48	GYPSY-201		1
Zebrafish	Gypsy	Barthez	Gy-Dr49	GYPSY52		4
Zebrafish	Gypsy	Barthez	Gy-Dr50	GYPSY54	2	3
Zebrafish	Gypsy	Barthez	Gy-Dr51	GYPSY59		3
Zebrafish	Gypsy	Barthez	Gy-Dr52	GYPSY6	2	7
Zebrafish	Gypsy	Barthez	Gy-Dr53	Gypsy60	1	1
Zebrafish	Gypsy	Barthez	Gy-Dr54	GYPSY63		5
Zebrafish	Gypsy	Barthez	Gy-Dr55	GYPSY74		
Zebrafish	Gypsy	Barthez	Gy-Dr56	GYPSY75		
Zebrafish	Gypsy	Barthez	Gy-Dr57	GYPSY8		7
Zebrafish	Gypsy	Barthez	Gy-Dr58	GYPSY85		1
Zebrafish	Gypsy	Barthez	Gy-Dr59	GYPSY9		1
Zebrafish	Gypsy	Barthez	Gy-Dr60	GYPSY94	1	4
Zebrafish	Gypsy	Osvaldo/Gmr	Gy-Dr61		1	
Zebrafish	Gypsy	Osvaldo/Gmr	Gy-Dr62	GYPSY-110	1	
Zebrafish	Gypsy	Osvaldo/Gmr	Gy-Dr63		1	
Zebrafish	Gypsy	Osvaldo/Gmr	Gy-Dr64	Gypsy121	1	
Zebrafish	Gypsy	Osvaldo/Gmr	Gy-Dr65	Gypsy124	1	2
Zebrafish	Gypsy	Osvaldo/Gmr	Gy-Dr66		2	4
Zebrafish	Gypsy	Osvaldo/Gmr	Gy-Dr67			1
Zebrafish	Gypsy	Osvaldo/Gmr	Gy-Dr68	GYPSY161		3
Zebrafish	Gypsy	Osvaldo/Gmr	Gy-Dr69	GYPSY-32		4
Zebrafish	Gypsy	Osvaldo/Gmr	Gy-Dr70	Gypsy-31	10	7
Zebrafish	Gypsy	Osvaldo/Gmr	Gy-Dr71	GYPSY87		3
Zebrafish	Gypsy	Osvaldo/Gmr	Gy-Dr72	Gypsy49	2	6
Zebrafish	Gypsy	Osvaldo/Gmr	Gy-Dr73	GYPSY-167	1	1
Zebrafish	Gypsy	Osvaldo/Gmr	Gy-Dr74		1	4
Zebrafish	Gypsy	Osvaldo/Gmr	Gy-Dr75		1	2
Zebrafish	Gypsy	Osvaldo/Gmr	Gy-Dr76		3	1
Zebrafish	Gypsy	Osvaldo/Gmr	Gy-Dr77			1
Zebrafish	Gypsy	Osvaldo/Gmr	Gy-Dr78		3	4

Zebrafish	Gypsy	Osvaldo/Gmr	Gy-Dr79	GYPSY-29	2	11
Zebrafish	Gypsy	Osvaldo/Gmr	Gy-Dr80	GYPSY122	1	3
Zebrafish	Gypsy	Osvaldo/Gmr	Gy-Dr81		4	3
Zebrafish	Gypsy	Osvaldo/Gmr	Gy-Dr82			
Zebrafish	Gypsy	Osvaldo/Gmr	Gy-Dr83	Gypsy90	4	6
Zebrafish	Gypsy	Osvaldo/Gmr	Gy-Dr84		1	1
Zebrafish	Gypsy	Osvaldo/Gmr	Gy-Dr85			3
Zebrafish	Gypsy	Osvaldo/Gmr	Gy-Dr86	GYPSY42	3	3
Zebrafish	Gypsy	Osvaldo/Gmr	Gy-Dr87	GYPSY139	26	27
Zebrafish	Gypsy	Osvaldo/Gmr	Gy-Dr88		28	26
Zebrafish	Gypsy	Osvaldo/Gmr	Gy-Dr89	Gypsy-116	4	2
Zebrafish	Gypsy	Osvaldo/Gmr	Gy-Dr90	gypsy119		
Zebrafish	Gypsy	Osvaldo/Gmr	Gy-Dr91	Gypsy120	1	5
Zebrafish	Gypsy	Osvaldo/Gmr	Gy-Dr92	GYPSY123	2	5
Zebrafish	Gypsy	Osvaldo/Gmr	Gy-Dr93	Gypsy-130	1	1
Zebrafish	Gypsy	Osvaldo/Gmr	Gy-Dr94	Gypsy131	1	1
Zebrafish	Gypsy	Osvaldo/Gmr	Gy-Dr95	Gypsy152	7	2
Zebrafish	Gypsy	Osvaldo/Gmr	Gy-Dr96	GYPSY155	1	
Zebrafish	Gypsy	Osvaldo/Gmr	Gy-Dr97	GYPSY-22		3
Zebrafish	Gypsy	Osvaldo/Gmr	Gy-Dr98	GYPSY-220	1	
Zebrafish	Gypsy	Osvaldo/Gmr	Gy-Dr99	GYPSY-221		1
Zebrafish	Gypsy	Osvaldo/Gmr	Gy-Dr100	GYPSY-24		7
Zebrafish	Gypsy	Osvaldo/Gmr	Gy-Dr101	GYPSY-25	2	1
Zebrafish	Gypsy	Osvaldo/Gmr	Gy-Dr102	GYPSY-26		2
Zebrafish	Gypsy	Osvaldo/Gmr	Gy-Dr103	GYPSY-27		2
Zebrafish	Gypsy	Osvaldo/Gmr	Gy-Dr104	GYPSY-30		6
Zebrafish	Gypsy	Osvaldo/Gmr	Gy-Dr105	GYPSY-33		2
Zebrafish	Gypsy	Osvaldo/Gmr	Gy-Dr106	GYPSY-34	6	6
Zebrafish	Gypsy	Osvaldo/Gmr	Gy-Dr107	Gypsy35	2	1
Zebrafish	Gypsy	Osvaldo/Gmr	Gy-Dr108	GYPSY37	1	1
Zebrafish	Gypsy	Osvaldo/Gmr	Gy-Dr109	GYPSY38		1
Zebrafish	Gypsy	Osvaldo/Gmr	Gy-Dr110	GYPSY39	6	6
Zebrafish	Gypsy	Osvaldo/Gmr	Gy-Dr111	Gypsy40	3	2

Zebrafish	Gypsy	Osvaldo/Gmr	Gy-Dr112	Gypsy41	4	11
Zebrafish	Gypsy	Osvaldo/Gmr	Gy-Dr113	Gypsy43	2	3
Zebrafish	Gypsy	Osvaldo/Gmr	Gy-Dr114	GYPSY45		
Zebrafish	Gypsy	Osvaldo/Gmr	Gy-Dr115	GYPSY46	3	3
Zebrafish	Gypsy	Osvaldo/Gmr	Gy-Dr116	GYPSY48		3
Zebrafish	Gypsy	Osvaldo/Gmr	Gy-Dr117	GYPSY7		6
Zebrafish	Gypsy	Osvaldo/Gmr	Gy-Dr118	GYPSY77		
Zebrafish	Gypsy	Osvaldo/Gmr	Gy-Dr119	Gypsy91	51	34
Zebrafish	Gypsy	Osvaldo/Gmr	Gy-Dr120	Gypsy47	6	6
Zebrafish	Gypsy	Osvaldo/Gmr	Gy-Dr121	GYPY119	1	
Zebrafish	Gypsy	Mag	Gy-Dr122		2	4
Zebrafish	Gypsy	Mag	Gy-Dr123	Gypsy55	3	
Zebrafish	Gypsy	Mag	Gy-Dr124			1
Zebrafish	Gypsy	Mag	Gy-Dr125	Gypsy92	4	5
Zebrafish	Gypsy	Mag	Gy-Dr126	Gypsy160	1	1
Zebrafish	Gypsy	Mag	Gy-Dr127	GYPSY86	2	2
Zebrafish	Gypsy	Mag	Gy-Dr128		2	2
Zebrafish	Gypsy	Mag	Gy-Dr129	Gypsy67	3	5
Zebrafish	Gypsy	Mag	Gy-Dr130			2
Zebrafish	Gypsy	Mag	Gy-Dr131	GYPSY-207	1	3
Zebrafish	Gypsy	Mag	Gy-Dr132			2
Zebrafish	Gypsy	Mag	Gy-Dr133	GYPSY153		8
Zebrafish	Gypsy	Mag	Gy-Dr134	GYPSY-111		
Zebrafish	Gypsy	Mag	Gy-Dr135	Gypsy141	3	2
Zebrafish	Gypsy	Mag	Gy-Dr136	Gypsy142	4	7
Zebrafish	Gypsy	Mag	Gy-Dr137	GYPSY143	2	2
Zebrafish	Gypsy	Mag	Gy-Dr138	GYPSY158	1	2
Zebrafish	Gypsy	Mag	Gy-Dr139	GYPSY163		1
Zebrafish	Gypsy	Mag	Gy-Dr140	GYPSY-179		
Zebrafish	Gypsy	Mag	Gy-Dr141	Gypsy-189	4	2
Zebrafish	Gypsy	Mag	Gy-Dr142	GYPSY-204		
Zebrafish	Gypsy	Mag	Gy-Dr143	GYPSY-217		1
Zebrafish	Gypsy	Mag	Gy-Dr144	GYPSY53	3	4

Zebrafish	Gypsy	Mag	Gy-Dr145	GYPSY58	2	2
Zebrafish	Gypsy	Mag	Gy-Dr146	Gypsy68	1	1
Zebrafish	Gypsy	Mag	Gy-Dr147	GYPSY73		1
Zebrafish	Gypsy	Mag	Gy-Dr148	GYPSY93	2	1
Zebrafish	Gypsy	Mag	Gy-Dr149	Gypsy95	1	2
Zebrafish	Gypsy	Skipper	Gy-Dr150	GYPSY146	1	1
Zebrafish	Gypsy	Skipper	Gy-Dr151	GYPSY57		1
Zebrafish	Gypsy	Skipper	Gy-Dr152	GYPSY65		3
Zebrafish	Gypsy	Skipper	Gy-Dr153	GYPSY78		
Zebrafish	Gypsy	Skipper-like	Gy-Dr154	GYPSY-188		2
Zebrafish	Gypsy	Skipper-like	Gy-Dr155	GYPSY106		5
Zebrafish	Gypsy	Skipper-like	Gy-Dr156	GYPSY149		1
Zebrafish	Gypsy	Skipper-like	Gy-Dr157	GYPSY151		1
Zebrafish	Gypsy	Skipper-like	Gy-Dr158	GYPSY-171	1	2
Zebrafish	Gypsy	Skipper-like	Gy-Dr159	GYPSY-186		
Zebrafish	Gypsy	Skipper-like	Gy-Dr160	GYPSY4		2
Zebrafish	Gypsy	Skipper-like	Gy-Dr161	GYPSY66		1
Zebrafish	Gypsy	V-clade	Gy-Dr162	GYPSY159	2	2
Zebrafish	Gypsy	V-clade	Gy-Dr163	GYPSY62	2	1
Zebrafish	Gypsy	V-clade	Gy-Dr164	GYPSY105	7	9
Zebrafish	Gypsy	V-clade	Gy-Dr165	GYPSY70	7	7
Zebrafish	Gypsy	V-clade	Gy-Dr166		1	1
Zebrafish	Gypsy	V-clade	Gy-Dr167		1	1
Zebrafish	Gypsy	V-clade	Gy-Dr168	GYPSY138	4	5
Zebrafish	Gypsy	V-clade	Gy-Dr169	GYPSY89	2	6
Zebrafish	Gypsy	V-clade	Gy-Dr170	GYPSY88	10	16
Zebrafish	Gypsy	V-clade	Gy-Dr171	Gypsy170	40	48
Zebrafish	Gypsy	V-clade	Gy-Dr172		1	3
Zebrafish	Gypsy	V-clade	Gy-Dr173			2
Zebrafish	Gypsy	V-clade	Gy-Dr174		1	2
Zebrafish	Gypsy	V-clade	Gy-Dr175	Gypsy-194	3	5
Zebrafish	Gypsy	V-clade	Gy-Dr176		8	9
Zebrafish	Gypsy	V-clade	Gy-Dr177	Gypsy-28b	10	15

Zebrafish	Gypsy	V-clade	Gy-Dr178	GYPSY-165	13	13
Zebrafish	Gypsy	V-clade	Gy-Dr179	Gypsydr2	37	67
Zebrafish	Gypsy	V-clade	Gy-Dr180	Gypsy101	3	4
Zebrafish	Gypsy	V-clade	Gy-Dr181	Gypsy107	3	4
Zebrafish	Gypsy	V-clade	Gy-Dr182	Gypsy-115	30	30
Zebrafish	Gypsy	V-clade	Gy-Dr183	GYPSY145		1
Zebrafish	Gypsy	V-clade	Gy-Dr184	GYPSY154		
Zebrafish	Gypsy	V-clade	Gy-Dr185	Gypsy164b	6	2
Zebrafish	Gypsy	V-clade	Gy-Dr186	Gypsy-168		
Zebrafish	Gypsy	V-clade	Gy-Dr187	Gypsy-173	8	10
Zebrafish	Gypsy	V-clade	Gy-Dr188	Gypsy69	4	4
Zebrafish	Gypsy	V-clade	Gy-Dr189	GYPSY72	1	2
Zebrafish	Gypsy	V-clade	Gy-Dr190	GYPSY76	3	4
Stickleback	BEL/PAO	Suzu	Bel-Ga1	Bel-7	3	3
Stickleback	BEL/PAO	Suzu	Bel-Ga2	Bel-10	1	
Stickleback	BEL/PAO	Sinbad	Bel-Ga3	Bel-11	1	2
Stickleback	BEL/PAO	Sinbad	Bel-Ga4	Bel-9		3
Stickleback	BEL/PAO	Sinbad	Bel-Ga5	Bel-8	2	1
Stickleback	BEL/PAO	Sinbad	Bel-Ga6	Bel-6	2	2
Stickleback	BEL/PAO	Sinbad	Bel-Ga7	Bel-1	1	1
Stickleback	BEL/PAO	Sinbad	Bel-Ga8	Bel-5	1	1
Stickleback	BEL/PAO	Sinbad	Bel-Ga9	Bel-2	1	1
Stickleback	BEL/PAO	Sinbad	Bel-Ga10		2	
Stickleback	BEL/PAO	Sinbad	Bel-Ga11		2	
Stickleback	Copia	Copia	Co-Ga1	Copia-2	2	4
Stickleback	Copia	Copia	Co-Ga2	Copia-3		2
Stickleback	Copia	Copia	Co-Ga3	Copia-1	1	1
Stickleback	ERV	Epsilon	ERV-Ga1			1
Stickleback	ERV	Epsilon	ERV-Ga2			1
Stickleback	ERV	Epsilon	ERV-Ga3			2

Stickleback	ERV	Epsilon	ERV-Ga4		1	
Stickleback	ERV	Epsilon	ERV-Ga5	Gypsy-52	2	5
Stickleback	ERV	Epsilon	ERV-Ga6	Gypsy-30	3	7
Stickleback	ERV	Epsilon	ERV-Ga7			1
Stickleback	ERV	Epsilon	ERV-Ga8		1	
Stickleback	ERV	Epsilon	ERV-Ga9			2
Stickleback	ERV	Epsilon	ERV-Ga10			1
Stickleback	ERV	Epsilon	ERV-Ga11	Gypsy-6	1	1
Stickleback	ERV	Epsilon	ERV-Ga12			1
Stickleback	ERV	Epsilon	ERV-Ga13			1
Stickleback	ERV	Epsilon	ERV-Ga14		1	
Stickleback	ERV	Epsilon	ERV-Ga15			1
Stickleback	ERV	Epsilon	ERV-Ga16		1	2
Stickleback	Gypsy	Barthez	Gy-Ga1	Gypsy-19	7	9
Stickleback	Gypsy	Barthez	Gy-Ga2	Gypsy-37	3	4
Stickleback	Gypsy	Barthez	Gy-Ga3	Gypsy-46	4	10
Stickleback	Gypsy	Barthez	Gy-Ga4	Gypsy-49	2	4
Stickleback	Gypsy	Barthez	Gy-Ga5	Gypsy-50		3
Stickleback	Gypsy	Barthez	Gy-Ga6		1	1
Stickleback	Gypsy	Barthez	Gy-Ga7		1	
Stickleback	Gypsy	Barthez	Gy-Ga8		1	1
Stickleback	Gypsy	Barthez	Gy-Ga9		1	1
Stickleback	Gypsy	Barthez	Gy-Ga10			1
Stickleback	Gypsy	CsRN1	Gy-Ga11	Gypsy-13	7	6
Stickleback	Gypsy	CsRN1	Gy-Ga12	Gypsy-2	1	2
Stickleback	Gypsy	Osvaldo/Gmr	Gy-Ga13	Gypsy-16	5	2
Stickleback	Gypsy	Osvaldo/Gmr	Gy-Ga14	Gypsy-18	1	
Stickleback	Gypsy	Osvaldo/Gmr	Gy-Ga15	Gypsy-21	1	1
Stickleback	Gypsy	Osvaldo/Gmr	Gy-Ga16	Gypsy-25	2	
Stickleback	Gypsy	Osvaldo/Gmr	Gy-Ga17	Gypsy-27	1	3
Stickleback	Gypsy	Osvaldo/Gmr	Gy-Ga18	Gypsy-3	1	1
Stickleback	Gypsy	Osvaldo/Gmr	Gy-Ga19	Gypsy-33	3	5

Stickleback	Gypsy	Osvaldo/Gmr	Gy-Ga20	Gypsy-34	1	
Stickleback	Gypsy	Osvaldo/Gmr	Gy-Ga21	Gypsy-4	4	4
Stickleback	Gypsy	Osvaldo/Gmr	Gy-Ga22	Gypsy-42	5	3
Stickleback	Gypsy	Osvaldo/Gmr	Gy-Ga23	Gypsy-45	7	1
Stickleback	Gypsy	Osvaldo/Gmr	Gy-Ga24	Gypsy-53	6	5
Stickleback	Gypsy	Osvaldo/Gmr	Gy-Ga25	Gypsy-54	2	3
Stickleback	Gypsy	Osvaldo/Gmr	Gy-Ga26	Gypsy-57	1	2
Stickleback	Gypsy	Osvaldo/Gmr	Gy-Ga27		2	2
Stickleback	Gypsy	Osvaldo/Gmr	Gy-Ga28		7	2
Stickleback	Gypsy	Osvaldo/Gmr	Gy-Ga29		1	
Stickleback	Gypsy	Osvaldo/Gmr	Gy-Ga30		1	1
Stickleback	Gypsy	Osvaldo/Gmr	Gy-Ga31			1
Stickleback	Gypsy	Osvaldo/Gmr	Gy-Ga32			1
Stickleback	Gypsy	Osvaldo/Gmr	Gy-Ga33		1	1
Stickleback	Gypsy	Osvaldo/Gmr	Gy-Ga34		1	
Stickleback	Gypsy	Mag	Gy-Ga35	Gypsy-17	2	
Stickleback	Gypsy	Mag	Gy-Ga36	Gypsy-20	1	2
Stickleback	Gypsy	Mag	Gy-Ga37	Gypsy-26	1	
Stickleback	Gypsy	Mag	Gy-Ga38	Gypsy-40	2	24
Stickleback	Gypsy	Mag	Gy-Ga39			1
Stickleback	Gypsy	Skipper	Gy-Ga40	Gypsy-56	4	4
Stickleback	Gypsy	Skipper-like	Gy-Ga41	Gypsy-22	1	1
Stickleback	Gypsy	Skipper-like	Gy-Ga42	Gypsy-23	1	1
Stickleback	Gypsy	V-clade	Gy-Ga43	Gypsy-10	8	7
Stickleback	Gypsy	V-clade	Gy-Ga44	Gypsy-12	3	4
Stickleback	Gypsy	V-clade	Gy-Ga45	Gypsy-24	22	22
Stickleback	Gypsy	V-clade	Gy-Ga46	Gypsy-28	2	5
Stickleback	Gypsy	V-clade	Gy-Ga47	Gypsy-29	1	1
Stickleback	Gypsy	V-clade	Gy-Ga48	Gypsy-35	2	7
Stickleback	Gypsy	V-clade	Gy-Ga49	Gypsy-38	4	5
Stickleback	Gypsy	V-clade	Gy-Ga50	Gypsy-41	3	3
Stickleback	Gypsy	V-clade	Gy-Ga51	Gypsy-43	3	4
Stickleback	Gypsy	V-clade	Gy-Ga52	Gypsy-47	12	10

Stickleback	Gypsy	V-clade	Gy-Ga53	Gypsy-5	1	1
Stickleback	Gypsy	V-clade	Gy-Ga54	Gypsy-55	7	12
Stickleback	Gypsy	V-clade	Gy-Ga55	Gypsy-7	2	4
Stickleback	Gypsy	V-clade	Gy-Ga56	Gypsy-8	5	9
Stickleback	Gypsy	V-clade	Gy-Ga57			1
Stickleback	Gypsy	V-clade	Gy-Ga58		1	1
Stickleback	Gypsy	V-clade	Gy-Ga59		1	
Stickleback	Gypsy	V-clade	Gy-Ga60		1	
Stickleback	Gypsy	V-clade	Gy-Ga61		2	3
Medaka	BEL/PAO	Suzu	Bel-Ol1		1	1
Medaka	BEL/PAO	Sinbad	Bel-Ol2			1
Medaka	BEL/PAO	Sinbad	Bel-Ol3			1
Medaka	BEL/PAO	Sinbad	Bel-Ol4			1
Medaka	BEL/PAO	Sinbad	Bel-Ol5			1
Medaka	BEL/PAO	PAO	Bel-Ol6			1
Medaka	Copia	Copia	Copia-Ol1			
Medaka	ERV	Epsilon	ERV-Ol1			
Medaka	Gypsy	Barthez	Gy-Ol1		1	2
Medaka	Gypsy	Barthez	Gy-Ol2			1
Medaka	Gypsy	Barthez	Gy-Ol3		1	1
Medaka	Gypsy	Barthez	Gy-Ol4			2
Medaka	Gypsy	Barthez	Gy-Ol5			1
Medaka	Gypsy	Barthez	Gy-Ol6			1
Medaka	Gypsy	Barthez	Gy-Ol7			1
Medaka	Gypsy	Barthez	Gy-Ol8			1
Medaka	Gypsy	Barthez	Gy-Ol9			1
Medaka	Gypsy	Barthez	Gy-Ol10			1
Medaka	Gypsy	Barthez	Gy-Ol11			2
Medaka	Gypsy	CsRN1	Gy-Ol12			1

Medaka	Gypsy	Osvaldo/Gmr	Gy-Ol13		1	1
Medaka	Gypsy	Osvaldo/Gmr	Gy-Ol14			1
Medaka	Gypsy	Osvaldo/Gmr	Gy-Ol15	GypsyOL		
Medaka	Gypsy	Mag	Gy-Ol16		1	1
Medaka	Gypsy	Skipper	Gy-Ol17		1	
Medaka	Gypsy	Skipper-like	Gy-Ol18			1
Medaka	Gypsy	Skipper-like	Gy-Ol19			1
Medaka	Gypsy	Skipper-like	Gy-Ol20			1
Medaka	Gypsy	V-clade	Gy-Ol21			2
Medaka	Gypsy	V-clade	Gy-Ol22		1	1
Medaka	Gypsy	V-clade	Gy-Ol23		1	1
Medaka	Gypsy	V-clade	Gy-Ol24		1	2
Medaka	Gypsy	V-clade	Gy-Ol25		2	**2**
Medaka	Gypsy	V-clade	Gy-Ol26			2
Medaka	Gypsy	V-clade	Gy-Ol27		1	3
Medaka	Gypsy	V-clade	Gy-Ol28			1
Medaka	Gypsy	V-clade	Gy-Ol29			2
Medaka	Gypsy	V-clade	Gy-Ol30			2
Medaka	Gypsy	V-clade	Gy-Ol31		1	2
Medaka	Gypsy	V-clade	Gy-Ol32			1
Medaka	Gypsy	V-clade	Gy-Ol33		1	1
Medaka	Gypsy	V-clade	Gy-Ol34			1
Medaka	Gypsy	V-clade	Gy-Ol35		1	
Medaka	Gypsy	V-clade	Gy-Ol36		1	
Medaka	Gypsy	V-clade	Gy-Ol37		1	
Tetraodon	Gypsy	Barthez	Gy-Tn1		1	1
Tetraodon	Gypsy	CsRN1	Gy-Tn2		1	2
Tetraodon	Gypsy	Osvaldo/Gmr	Gy-Tn3			1
Tetraodon	Gypsy	Mag	Gy-Tn4			1
Tetraodon	Gypsy	Skipper	Gy-Tn5			1
Tetraodon	Gypsy	V-clade	Gy-Tn6		1	1
Tetraodon	Gypsy	V-clade	Gy-Tn7			1

Tetraodon	Gypsy	V-clade	Gy-Tn8		1	1
