# Supplementary material for: The contribution of transposable elements to size variations between four teleost genomes
Source: Mob DNA. 2016 Feb 9;7:4. doi: 10.1186/s13100-016-0059-7 (PMC4746887; doi:10.1186/s13100-016-0059-7)
Supplement: Additional file 4: Table S4. — Abundance of retrotransposons in teleost genomes. (PDF 181 kb) [file 13100_2016_59_MOESM4_ESM.pdf]

Additional file 4: Table S4. Abundance of retrotransposons in teleost genomes.

	Group	Zebrafish			Medaka			Stickleback			Tetraodon		
		Copy number	bp	%	Copy number	bp	%	Copy number	bp	%	Copy number	bp	%
LINE													
	L1	35022	16959165	1.24	14483	5024695	0.58	2928	879026	0.19	493	353257	0.10
	L2	52418	22033143	1.61	42789	13668469	1.57	17494	5521262	1.20	3437	596473	0.17
	RTE	7779	3685893	0.27	27144	7259048	0.84	3352	1448452	0.31	2979	1144459	0.32
	Rex-Babar	15437	5304055	0.39	14317	4468815	0.51	10614	3174280	0.69	6316	2000859	0.56
	Penelope	16772	1547509	0.11	6447	1109295	0.13	132	249508	0.05	4698	1026614	0.29
	R2	337	211796	0.02	281	141243	0.02	13	15754	0.00	1106	680569	0.19
	I	5123	2698161	0.20	237	96133	0.01	672	187804	0.04	356	136005	0.04
	CR1				1260	559681	0.06						
	DRE				243	100120	0.01						
	Dong-R4				3973	1921282	0.22						
	Proto2				904	361074	0.04	151	61655	0.01			
	R1				247	30783	0.00						
	Jockey							240	71180	0.02			
LTR													
	Copia	630	923998	0.07	4368	890074	0.10	135	117619	0.03	310	122485	0.03
	DIRS	7923	14496813	1.06				328	77200	0.02	262	170477	0.05
	ERV	31405	9086741	0.66	1155	703982	0.08	27464	4435919	0.96	5770	634024	0.18
	Gypsy	73503	33235984	2.42	37226	10787599	1.24	26182	8544862	1.85	5844	4433833	1.24
	Ngaro	35017	12247395	0.89	27660	5608149	0.65	2040	502122	0.11	2292	438828	0.12
	BEL/PAO	4266	3311671	0.24	2116	958993	0.11	2828	1115246	0.24	179	53208	0.01
SINE		136879	30638751	2.24	30578	4792569	0.68	11523	2971330	0.67	1498	257342	0.09
