# Supplementary figures and images for: The contribution of transposable elements to size variations between four teleost genomes
Source: Mob DNA. 2016 Feb 9;7:4. doi: 10.1186/s13100-016-0059-7 (PMC4746887; doi:10.1186/s13100-016-0059-7)

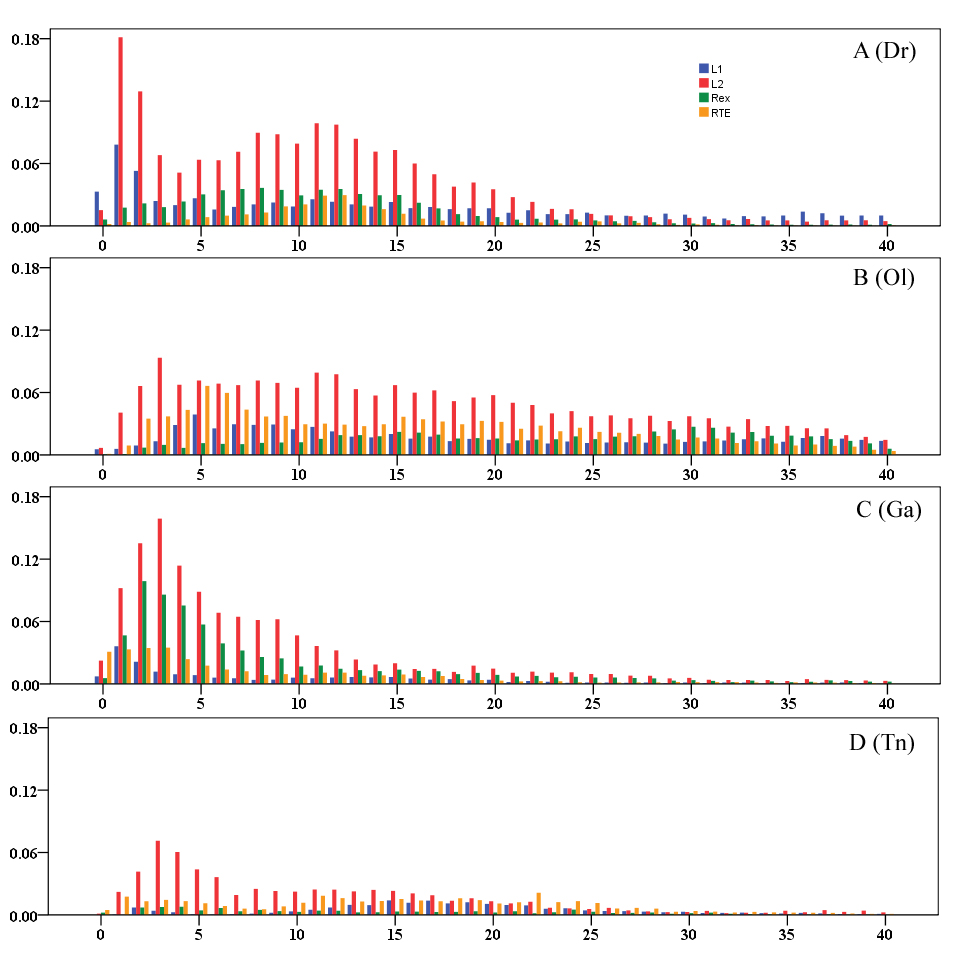

Supplement: Additional file 5: Figure S1. — Divergence distribution of the major clades of LINEs in the zebrafish (A), medaka (B), stickleback (C), and tetraodon (D) genomes. The x-axis represents the substitution rate from consensus sequences (%), and the y-axis represents the percentage of the genome comprised of repeat classes (%). (JPG 285 kb) [file 13100_2016_59_MOESM5_ESM.jpg]

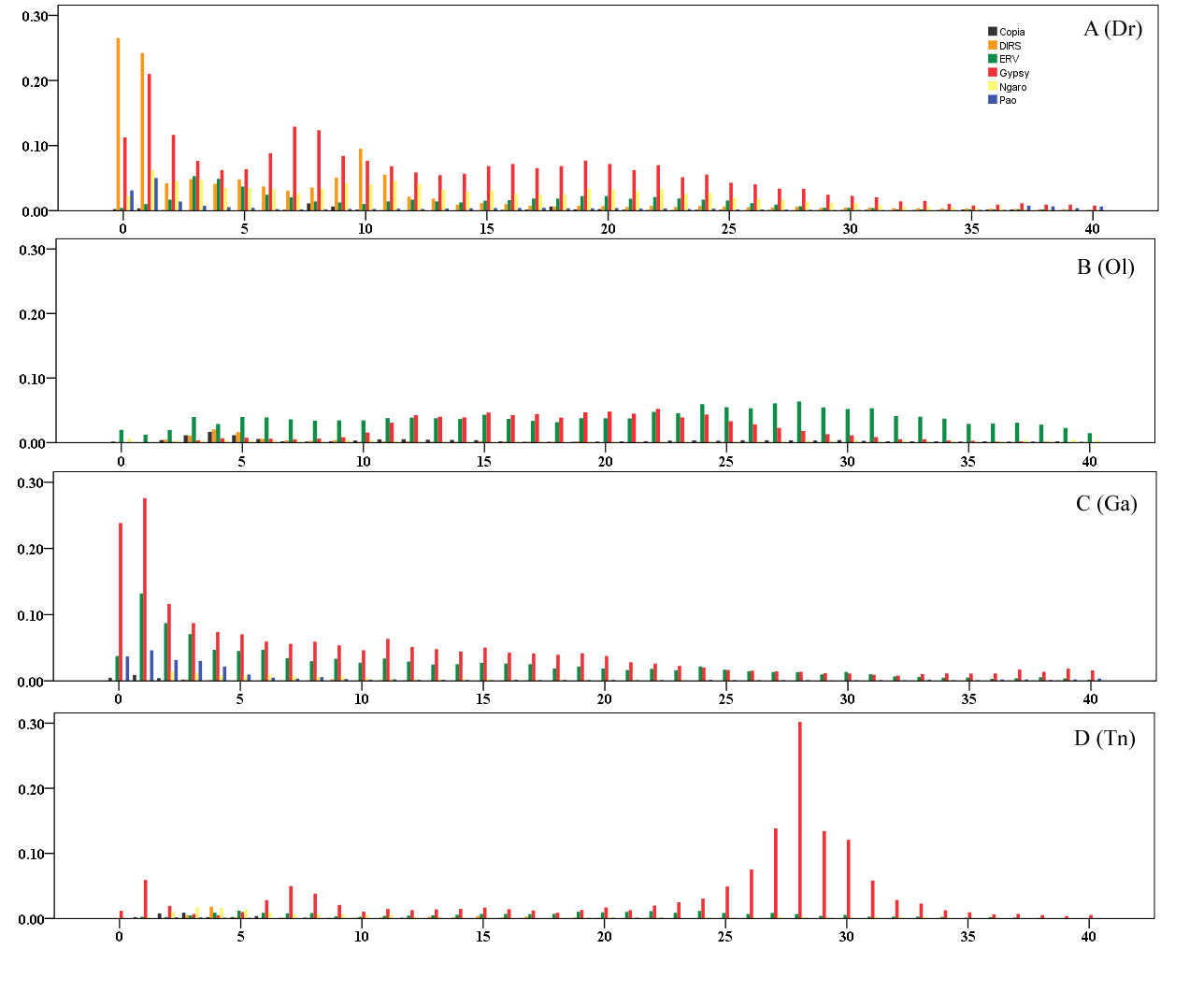

Supplement: Additional file 6: Figure S2. — Divergence distribution of the major groups of LTRs in the zebrafish (A), medaka (B), stickleback (C), and tetraodon (D) genomes. The x-axis represents the substitution rate from consensus sequences (%), and the y-axis represents the percentage of the genome comprised of repeat classes (%). (JPG 312 kb) [file 13100_2016_59_MOESM6_ESM.jpg]
